# TORC1 and PKA activity towards ribosome biogenesis oscillates in synchrony with the budding yeast cell cycle

**DOI:** 10.1242/jcs.260378

**Published:** 2022-09-28

**Authors:** Paolo Guerra, Luc-Alban P. E. Vuillemenot, Yulan B. van Oppen, Marije Been, Andreas Milias-Argeitis

**Affiliations:** Molecular Systems Biology, Groningen Biomolecular Sciences & Biotechnology Institute, University of Groningen, 9747 AG Groningen, The Netherlands

**Keywords:** PKA signaling, TOR signaling, TORC1, Cell growth, Ribosome biogenesis, Single-cell microscopy

## Abstract

Recent studies have revealed that the growth rate of budding yeast and mammalian cells varies during the cell cycle. By linking a multitude of signals to cell growth, the highly conserved target of rapamycin complex 1 (TORC1) and protein kinase A (PKA) pathways are prime candidates for mediating the dynamic coupling between growth and division. However, measurements of TORC1 and PKA activity during the cell cycle are still lacking. By following the localization dynamics of two TORC1 and PKA targets via time-lapse microscopy in hundreds of yeast (*Saccharomyces cerevisiae*) cells, we found that the activity of these pathways towards ribosome biogenesis fluctuates in synchrony with the cell cycle even under constant external conditions. Analysis of the effects of mutations of upstream TORC1 and PKA regulators suggests that internal metabolic signals partially mediate these activity changes. Our study reveals a new aspect of TORC1 and PKA signaling, which will be important for understanding growth regulation during the cell cycle.

## Teaser

**Highlighted Article:** Single-cell time-lapse microscopy of budding yeast reveals oscillations in the activity of TORC1 and PKA signaling pathways under constant external conditions.

## INTRODUCTION

Cell growth, the collection of processes through which cells accumulate biomass and increase in size, is essential for progression through the cell division cycle (Jorgensen and Tyers, 2004). However, although it has been well established that the cell cycle involves a complex temporal interplay of many different components, the prevailing notion has been that growth has a much lower temporal complexity, taking place at a constant or exponential rate throughout the cell cycle (Bryan et al., 2012; Elliott et al., 1979; Elliott and McLaughlin, 1978; Shulman et al., 1973; Di Talia et al., 2007). This notion, which is mostly supported by experimental evidence produced decades ago, has been challenged by studies with the model eukaryote *Saccharomyces cerevisiae* (budding yeast). As these works demonstrated, the volume increase rate of budding yeast cells displays a distinct oscillatory pattern during the cell cycle, with maxima during G1 and G2 and minima around the moment of budding and in mitosis (Cookson et al., 2010; Ferrezuelo et al., 2012). Furthermore, budding yeast cell density has been shown to reach a maximum prior to bud formation (Bryan et al., 2010), and autonomous growth rate oscillations coupled to the cell cycle of mammalian cells have also been reported recently (Liu et al., 2020). Collectively, these studies have shown that temporal dynamics of cell growth appears to be more complex than was previously thought.

The target of rapamycin (TOR) and protein kinase A (PKA) pathways are the two main, evolutionarily conserved regulators of cell growth in budding yeast, coupling carbon and nitrogen availability, internal metabolic signals and the presence of noxious stressors to several anabolic and catabolic processes that ultimately impact cell growth and division (Conrad et al., 2014; De Virgilio and Loewith, 2006; Filteau et al., 2015; González and Hall, 2017; Loewith and Hall, 2011; Zurita-Martinez and Cardenas, 2005). Importantly, TOR complex 1 (TORC1) and PKA together regulate ribosome biogenesis and protein synthesis (Jorgensen et al., 2004; Lippman and Broach, 2009; Martin et al., 2004; Powers and Walter, 1999; Urban et al., 2007; Yerlikaya et al., 2016), two of the most resource-intensive anabolic processes necessary for biomass accumulation and cell cycle progression (Kafri et al., 2016; Warner, 1999). TORC1 and PKA phosphorylate highly overlapping sets of targets involved in the regulation of ribosomal proteins (RPs), ribosome biogenesis (Ribi) factors and ribosomal RNAs via all three RNA polymerases (Soulard et al., 2010; Wullschleger et al., 2006). By regulating ribosome biogenesis, TORC1 (and likely PKA) also controls cytoplasmic crowding and density (Delarue et al., 2018; Neurohr and Amon, 2020). For this reason, these two signaling pathways could be involved in the generation of the non-monotonic growth rate dynamics observed during the cell cycle.

Some connections of TORC1 and PKA with the budding yeast cell cycle are known; besides their involvement in ribosome biogenesis, TORC1 and PKA have been implicated in the regulation of the G1/S transition by regulating the abundance of the early G1 cyclin Cln3 (Barbet et al., 1996; Hall et al., 1998; Mizunuma et al., 2013; Polymenis and Schmidt, 1997). They further antagonize the activity of the Rim15 kinase, which controls entry to G0 (Pedruzzi et al., 2003; Reinders et al., 1998; Schneper et al., 2004; Wanke et al., 2005). TORC1 also promotes the G1/S transition by stimulating the degradation of the cyclin-dependent kinase (CDK) inhibitor Sic1 (Moreno-Torres et al., 2017, 2015; Zinzalla et al., 2007), and is also involved in the regulation of G2/M transition (Nakashima et al., 2008; Tran et al., 2010). Furthermore, polarized growth of yeast cells induced by mating pheromone or apical bud growth in cells deficient in G1/S inhibitor degradation has been shown to suppress TORC1 activity towards protein synthesis and ribosome biogenesis (Goranov et al., 2013). Beyond yeast, mammalian TORC1 (mTORC1) is also repressed during mitosis (Odle et al., 2020). On the other hand, measurements of key downstream processes, such as ribosome biogenesis in yeast, have provided contradictory results, with different works proposing that ribosomal protein synthesis proceeds at a constant or exponentially increasing rate during the cell cycle (Elliott et al., 1979; Shulman et al., 1973). Therefore, despite evidence suggesting an interaction of TORC1 and PKA with the cell cycle, we still do not know the activity dynamics of these pathways during the cell cycle, as cell cycle-resolved measurements of TORC1 and/or PKA activity, both in yeast and higher eukaryotes are missing.

Motivated by the observations of growth rate and cell density oscillations, as well as the modulation of growth signaling activity by changes in cell morphology, we sought to investigate whether the activities of TORC1 and/or PKA pathways oscillate during the yeast cell cycle. Such a study is challenging to carry out with bulk methods (e.g. western blotting or mass spectrometry), which require synchronous cell populations; arrest-and-release techniques can perturb TORC1 and PKA activity, whereas less invasive approaches, such as centrifugal elutriation, might miss changes in pathway activity due to limited time resolution and/or population synchrony.

To avoid any potential artifacts of population-level methods, we employed single-cell time-lapse fluorescence microscopy of unperturbed dividing cells. This approach does not require synchronization while offering the possibility to align single-cell traces at different points during the cell cycle, and thus obtain a clear view of cell cycle-regulated processes at high time resolution. Given the central role of ribosomes in biomass accumulation (Lange and Heijnen, 2001), we focused on the ribosome biogenesis (Ribi) branch downstream of TORC1 and PKA and studied both pathways together due to the high degree of overlap of TORC1 and PKA targets in the Ribi network. To monitor TORC1 and PKA activity via microscopy, we screened transcriptional regulators of RP and Ribi expression whose nuclear localization responded to TORC1 and PKA activity and concluded that the nuclear-to-cytosolic ratio of the Sfp1 activator and Tod6 repressor can serve as a sensitive and fast TORC1 and PKA activity readouts. The localization dynamics of Sfp1 and Tod6 during unperturbed growth suggested that TORC1 and PKA activities towards ribosome biogenesis oscillate during the cell cycle, showing a maximum during G1 and a minimum at budding and late mitosis. Analysis of mutants suggested that upstream nutrient-sensing regulators of TORC1 and PKA are part of the mechanism that generates the activity oscillations. Finally, single-cell observations of two fluorescently tagged ribosomal proteins showed that the RP synthesis rate displays strong oscillations which reflect the TORC1 and PKA activity pattern. Our findings demonstrate that the activity of these central growth control pathways is temporally organized and tightly coordinated with the cell cycle. This new aspect of TORC1 and PKA signaling will be important for understanding the full scope of cellular activities regulated by these pathways and the mechanisms that couple growth and cell cycle progression.

## RESULTS

### Sfp1 and Tod6 localization is a sensitive and fast reporter of TORC1 and PKA activity

To follow TORC1 and PKA activity in the ribosome biogenesis branch, we searched for TORC1 and PKA targets whose signaling-dependent localization could be monitored via fluorescence time-lapse microscopy, focusing on transcriptional regulators of ribosome biogenesis whose intracellular localization is regulated by TORC1 and/or PKA. A member of this group is the split zinc-finger protein Sfp1 (a functional analog of the c-Myc proto-oncogene; Cook and Tyers, 2007; Lempiäinen et al., 2009), a direct TORC1 substrate and central activator of hundreds of RP and Ribi genes in response to nutrient and stress (Albert et al., 2019; Reja et al., 2015). Previous studies exploited the fact that the nuclear localization of Sfp1 is regulated by both TORC1 and PKA (Goranov et al., 2013; Jorgensen et al., 2004; Lempiäinen et al., 2009; Marion et al., 2004) and used it as a readout of TORC1 and PKA activity (Kingsbury and Cardenas, 2016; Saad et al., 2017; Singh and Tyers, 2009). Phosphorylation of Sfp1 by TORC1 increases the nuclear concentration of the protein and its binding to RP promoters (Albert et al., 2019; Jorgensen et al., 2004; Marion et al., 2004), whereas active PKA promotes the nuclear accumulation of Sfp1 (Marion et al., 2004; Singh and Tyers, 2009).

**Fig. 1.**
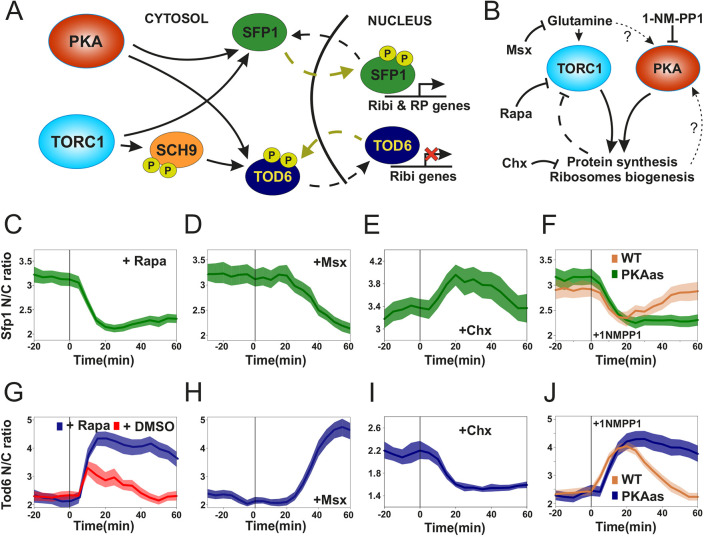
**Sfp1 and Tod6 localization is a sensitive and fast reporter of TORC1 and PKA activity.** (A) TORC1 and PKA regulate the phosphorylation and localization of Sfp1 and Tod6. (B) Schematic representation and summary of TORC1 and PKA chemical perturbations used in this work. (C–F) Sfp1 localization dynamics in response to perturbations shown in B (see Materials and Methods for details). Rapamycin was dissolved in minimal medium to avoid the strong effect of DMSO (see Materials and Methods). Inhibitors were added at time *t*=0 (vertical line). Bands denote the 95% confidence interval of the mean. *n*=60 in C, *n*=56 in D, *n*=56 in E, *n*=50 (PKAas) and *n*=39 (WT) in F. (G–J) Tod6 localization dynamics in response to perturbations shown in B (see Materials and Methods for details). Bands denote the 95% confidence interval of the mean. *n*=61 (treatment) and *n*=57 (control) in G, *n*=75 in H, *n*=56 in I, *n*=63 (PKAas) and n=66 (WT) in J.

Given the lack of a ‘gold standard’ for monitoring the activity of TORC1 and PKA via microscopy, we also sought a TORC1 and PKA readout complementary to Sfp1, so that one readout could be validated against the other. We therefore screened fluorescent fusions of three repressors of Ribi expression, Tod6, Dot6 and Stb3 (Huber et al., 2011, 2009; Liko et al., 2007, 2010), as well as the RP repressor Crf1 (Martin et al., 2004). The latter was undetectable via microscopy. Fluorescently tagged Stb3 was visible (Fig. S1), but was almost fully cytosolic under normal growth, which would compromise the precise quantification of variations in its nuclear-to-cytosolic (N/C) ratio during the cell cycle. Dot6 and Tod6 were both visible and showed an intermediate N/C ratio (Fig. S1). Tod6 and Dot6 are Myb-like helix-turn-helix (HTH) repressors of Ribi expression (Huber et al., 2011, 2009). They are directly phosphorylated and deactivated by the Sch9 kinase (itself a direct target of TORC1; Urban et al., 2007) and PKA at multiple Sch9 and PKA consensus sites (R[R/K]xS) (Huber et al., 2011, 2009). Contrary to Sfp1, phosphorylation of Tod6 and Dot6 is thought to promote their nuclear exit, although the evidence for this regulation remains circumstantial (Huber et al., 2011, 2009). It is also unclear how Tod6 and Dot6 localization responds to perturbations of TORC1 and PKA pathways. Fig. 1A summarizes the current model of Sfp1 and Tod6 regulation by TORC1 and PKA (Dot6 is not shown, but its regulation is similar to that of Tod6).

To monitor the nuclear localization of Sfp1, Tod6 and Dot6 in live cells, we tagged the Sfp1 and Tod6 with a pH-stable tandem GFP (Roberts et al., 2016) and Dot6 with mNeonGreen (mNG). We then quantified the fluorescent protein N/C ratio in cells carrying a fluorescently tagged histone (Hta2–mRFP) as a nuclear marker (see Materials and Methods and Fig. S1). C-terminal tagging of Sfp1 has been used in several past studies (Goranov et al., 2013; Jorgensen et al., 2004; Kingsbury and Cardenas, 2016; Lempiäinen et al., 2009; Marion et al., 2004; Saad et al., 2017; Singh and Tyers, 2009), and we verified that our tag causes just a minor increase in the mother doubling time, and no changes in cell size (Fig. S1). C-terminal tagging of Tod6 and Dot6 has also been used for monitoring the localization of these proteins via microscopy, although to a lesser extent (Granados et al., 2018; Santos et al., 2019). We verified that the tagging of Tod6 had no effect on the doubling time or cell size (Fig. S1). However, Dot6–mNG cells had a significantly shorter doubling time and a slightly larger size than the wild type (Fig. S1). These results suggest that the function of Sfp1 and Tod6 is not altered significantly by tagging, but the tagging of Do6 could compromise the function of this Ribi repressor.

To investigate whether changes in the N/C ratio of Sfp1, Tod6 and Dot6 can be used as a dynamic readout of TORC1 and PKA activity, we monitored Sfp1, Tod6 and Dot6 localization dynamics in response to acute perturbations of TORC1 and PKA upon treatment with rapamycin, methionine sulfoximine, cycloheximide and the bulky ATP-analog 1NM-PP1 (Fig. 1B). Methionine sulfoximine (MSX) is a tight-binding and specific inhibitor of glutamine synthetase, which causes the depletion of intracellular glutamine (a key metabolic input to TORC1; Stracka et al., 2014; Ukai et al., 2018), inhibits growth and causes a strong reduction in TORC1 activity towards nitrogen catabolite repression (Crespo et al., 2002; Stracka et al., 2014). Cycloheximide (CHX) blocks translation, whereas 1-NM-PP1 is a specific inhibitor of kinases carrying a point mutation that enlarges their ATP-binding pocket (Bishop et al., 2000).

In agreement with previous reports (Jorgensen et al., 2004; Marion et al., 2004), Sfp1 moved rapidly out of the nucleus upon inhibition of TORC1 with rapamycin (Fig. 1C; Fig. S1). Upon MSX treatment, we observed a decrease in the Sfp1 N/C ratio within 20 min, in agreement with the reported timescale for the depletion of the glutamine pool (Fig. 1D; Fig. S1) (Crespo et al., 2002). CHX treatment was previously reported to cause a transient increase in TORC1 activity (Jorgensen et al., 2004; Lempiäinen et al., 2009; Santos et al., 2019; Urban et al., 2007) via a still-unexplored negative-feedback mechanism (Fig. 1B; Fig. S1) (Eltschinger and Loewith, 2016). In line with this evidence, we also observed an increase in the Sfp1 N/C ratio after the addition of CHX (Fig. 1E; Fig. S1). Although rapamycin specifically inhibits TORC1, it should be noted that both MSX and CHX cause large metabolic rearrangements which could also alter PKA activity. To specifically inhibit PKA, we mutated its three kinase subunits (Tpk1, Tpk2 and Tpk3), rendering them sensitive to 1-NM-PP1 (PKAas) (Zaman et al., 2009). Shortly after the addition of 1-NM-PP1 to PKAas cells, we observed a decrease in the N/C ratio of Sfp1 with dynamics and magnitude similar to the rapamycin response, demonstrating that PKA is directly involved in the regulation of Sfp1 localization (Fig. 1F; Fig. S1). These results collectively suggest that Sfp1 localization reports dynamic changes in TORC1 and PKA activity.

Given that active TORC1 and/or PKA are thought to keep Tod6 and Dot6 (transcriptional repressors) out of the nucleus, we anticipated Tod6 and Dot6 nuclear localization to show an inverse relationship with TORC1 and PKA activity (Fig. 1A,B). We indeed observed a marked increase in the Tod6 and Dot6 nuclear localization after inhibition of TORC1 with rapamycin (Fig. 1G; Fig. S1). Treating cells with MSX also caused an increase in the Tod6 and Dot6 N/C ratio with dynamics mirroring the Sfp1 response (Fig. 1H; Fig. S1). In line with the Sfp1 results and with recently reported observations (Santos et al., 2019), CHX treatment also generated a drop in the Tod6 N/C ratio (Fig. 1I; Fig. S1), which is consistent with a transient increase in TORC1 activity. The CHX response of Dot6 was weaker than the Tod6 response because Dot6 is already less nuclear than Tod6 (compare Fig. 1G–J with Fig. S1G). Finally, inhibition of PKA by addition of 1-NM-PP1 to PKAas cells caused a fast and strong increase in Tod6 nuclear concentration, similar to what was seen with rapamycin treatment (Fig. 1J; Fig. S1). These results suggest that the nuclear localization of Tod6 and Dot6 is inversely related to TORC1 and PKA activity.

Collectively, our perturbation experiments show that Sfp1, Tod6 and Dot6 respond sensitively, quickly and on very similar timescales to abrupt changes in TORC1 and PKA activity. They could therefore serve as good proxies of TORC1 and PKA activity during an unperturbed cell cycle.

### Sfp1 and Tod6 localization oscillates in synchrony with the cell cycle

**Fig. 2.**
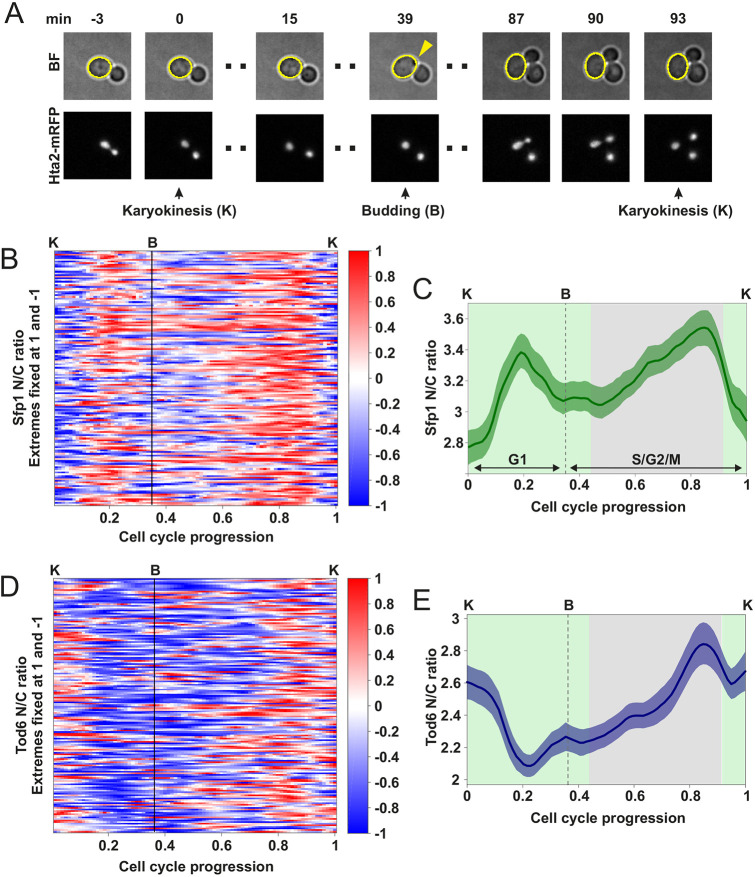
**Sfp1 and Tod6 localization oscillates in synchrony with the cell cycle.** (A) Cell cycle events used to manually divide N/C ratio time series into segments corresponding to distinct cell cycles. Karyokinesis (K) was detected with a nuclear fluorescent marker (Hta2–mRFP), and bud appearance (B) corresponded to the earliest moment of a visually detectable membrane deformation in brightfield (BF) cell images (yellow arrow). Single-cell N/C ratio traces corresponding to individual cell cycles were aligned at three points: a first karyokinesis, bud appearance and the subsequent karyokinesis. Image size 9.6 μm × 9.6 μm. (B) Heatmap of Sfp1 N/C ratio in individual cell cycles (*n*=149). Single-cell traces of Sfp1 N/C ratio were aligned and interpolated as described in Fig. 2A and in the Materials and Methods. For each cell cycle, the Sfp1 N/C ratio was normalized by assigning its maximum to 1 and its minimum to −1, to facilitate the identification of peaks and troughs. (C) Average Sfp1 N/C ratio dynamics for the cells shown in B. The averages were calculated without normalization of the single-cell data. The bands denote the 95% confidence interval of the mean. The average Sfp1 N/C ratio profile showed amplitudes in the order of ±10% around the mean, although single-cell trajectories typically display an even larger amplitude. Sfp1–mNeonGreen showed the same oscillatory pattern (Fig. S4D), suggesting that this behavior is not tag-specific. (D) Heatmap of Tod6 N/C ratio in individual cell cycles (*n*=161). Single-cell traces of Tod6 N/C ratio were processed in the same way as Sfp1 traces (B). (E) Average Tod6 N/C ratio dynamics for the cells shown in D, calculated similarly to the Sfp1 average (C). The average Tod6 N/C ratio profile showed amplitudes in the order of ±10% around the mean, although single-cell trajectories typically display an even larger amplitude. Similar to what was seen for Sfp1, Tod6-mNeonGreen localization dynamics showed the same oscillatory pattern (Fig. S5B). In C and E, the green background indicates the intervals in which the Sfp1 and Tod6 localization patterns are inverses of each other, whereas the gray background indicates the interval in which the Sfp1 and Tod6 patterns show the same behavior.

Having established Sfp1, Tod6 and Dot6 localization as a live-cell readout for TORC1 and PKA activity, we sought to investigate how the activity of these pathways might vary during an unperturbed cell cycle by monitoring the N/C ratio dynamics of these three proteins in mother cells grown under constant nutrient conditions and imaged for several hours at high temporal resolution (5 min). Using Hta2–mRFP as a nuclear marker, we quantified the Sfp1, Tod6 and Dot6 N/C ratio in single cells over several cell cycles. As cell cycle indicators, we recorded the earliest moment of bud appearance and karyokinesis, indicated by the split of nuclei labeled with Hta2–mRFP (see Materials and Methods; Fig. 2A; Fig. S2). In the nutrient conditions used, cytokinesis follows karyokinesis after ∼5 min (Garmendia-Torres et al., 2018), whereas the appearance of the bud implies the end of G1. Therefore, the interval between karyokinesis and bud appearance can be used to approximately locate the G1 phase of the cell cycle, and the interval between budding and karyokinesis roughly corresponds to S/G2/M (see Materials and Methods). By aligning the individual cell cycle trajectories at karyokinesis and bud appearance, we could analyze the average localization trends on a common (relative) time axis (see Materials and Methods).

We observed oscillations in the Sfp1–GFP N/C ratio of mother cells, both at the single-cell and population levels (Fig. 2B,C). Sfp1 nuclear localization displayed maxima in G1 and in G2/M, and minima at budding and karyokinesis (Fig. 2B,C). To determine more precisely the timing of the G1 maximum, we imaged Sfp1–GFP in mother cells carrying an mCherry-tagged copy of the Whi5 transcriptional repressor. Whi5 enters the nucleus shortly before cytokinesis and exits in the subsequent G1 phase after cells pass through the G1 checkpoint known as ‘Start’ (Costanzo et al., 2004). By calculating the cross-correlation (a measure of similarity) of the Whi5–mCherry and Sfp1–GFP time series in individual cells, we observed that the average cross-correlation is maximized at a negative time delay of ∼5–10 min, implying that the Whi5 localization peak precedes the Sfp1 peak by that amount of time (Fig. S2E). The negative cross-correlation at a positive time delay of ∼10 min also reflects the fact that the minimum of the Sfp1 N/C ratio at mitosis appears before the Whi5 localization peak. Tod6–GFP also displayed oscillations in localization with a minimum in G1, an increase through S/G2/M, a peak in late G2/M and high N/C ratio through karyokinesis (Fig. 2D,E). The Dot6–mNG N/C ratio showed a similar minimum in G1 and a maximum through karyokinesis but increased later than Tod6 in G2/M (Fig. S2). The differences in the localization patterns of Tod6 and Dot6 in S/G2/M (as well as their difference in the average N/C ratio) suggest that these proteins have diverged in terms of their regulation (Lippman and Broach, 2009). On a practical level, the amplitude of the Dot6 N/C ratio oscillations was smaller than that of Tod6, which was expected given that Dot6 is more cytosolic than Tod6 (Fig. S1B). This fact, together with the changes in doubling time and volume caused by Dot6 tagging (see results in the previous section) led us to exclude Dot6 from subsequent analyses of mutants.

The inverse patterns of Sfp1 compared with Tod6 and Dot6 localization during karyokinesis, G1 and early S/G2 (green shaded areas in Fig. 2C,E) are consistent with the inverse responses observed in the chemical perturbations of TORC1 and PKA, showing that the localization of these proteins is likely controlled by TORC1 and PKA in those phases. The localization patterns indicate a TORC1 and PKA activity peak in G1 and lower activity around karyokinesis and early S/G2. On the other hand, Sfp1 and Tod6 showed a common increase in average nuclear localization during G2/M, which needed closer examination.

### Changes in Tod6 localization during G2/M are not caused by TORC1 and PKA activity

**Fig. 3.**
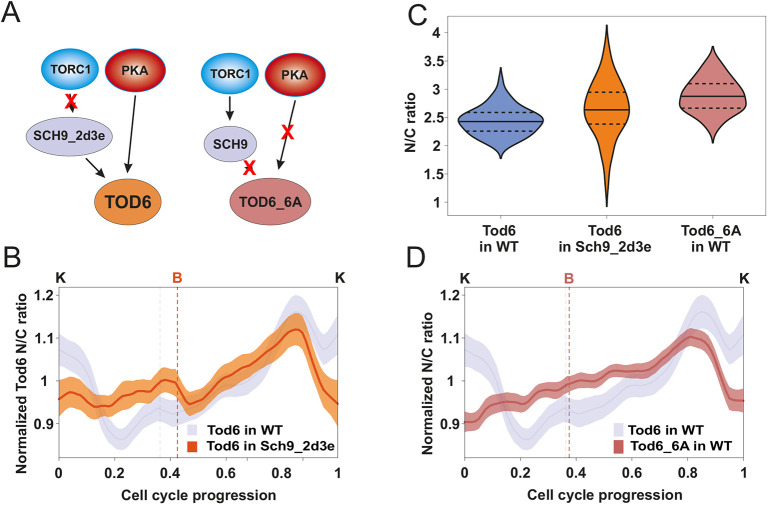
**Changes in Tod6 localization during G2/M are not caused by TORC1 or PKA activity.** (A) Altered regulation of Sch9_2d3e and Tod_6A with respect to wild type (WT). Left, phosphomimetic mutations at the TORC1-dependent Sch9 sites render Sch9 independent from TORC1. Right, alanine substitutions at the six Tod6 sites targeted by Sch9 and PKA decouple Tod6 regulation from TORC1 and PKA. (B) Average Tod6 N/C ratio dynamics in the Sch9_2d3e background (*n*=103) compared to the wild-type dynamics (note: wild-type data same as in Fig. 2E, repeated here to facilitate visual comparison). The averages were calculated after normalizing each cell cycle trace by its mean since the average localization of Tod6 differs in the two strains. Cell cycle traces were interpolated and aligned as described in Fig. 2A. Bands denote the 95% confidence interval for the mean. (C) Tod6 N/C ratio distributions in the wild-type (*n*=70), and the Sch9_2d3e (*n*=63) and Tod6_6A (*n*=70) mutants. Median (continuous line) and 25th and 75th percentiles (dashed lines) are displayed. Statistical comparison, two-tailed Mann–Whitney test: Sch9_2d3e to WT *P*=8.5×10^−4^, effect size (rank-biserial correlation) *r*=0.39; Tod6_6A to WT *P*=2.5×10^−14^, *r*=0.78; Tod6_6A to Sch9_2d3e, *P*=0.002, *r*=0.31. (D) Average Tod6_6A N/C ratio dynamics (*n*=109) compared to the wild-type Tod6 dynamics (note: wild-type data same as in Fig. 2E, repeated here to facilitate visual comparison). Calculation of averages and cell cycle trace processing were carried out as in B.

To investigate whether the localization of Tod6 in G2/M is controlled by TORC1 or PKA, we turned to the analysis of Sfp1 and Tod6 localization in mutants, supported by a new method for the statistical comparison of groups of single-cell time series (see Materials and Methods). We first focused on Sch9, the kinase that relays the signal from TORC1 to Tod6 (Huber et al., 2011). We replaced the endogenous Sch9 protein by a mutant in which the residues phosphorylated by TORC1 were replaced with amino acids that mimic constitutive phosphorylation (Sch9_2d3e) (Urban et al., 2007). In this mutant strain, Sch9 regulation is decoupled from TORC1, meaning that inputs from Sch9 to Tod6 are no longer associated with changes in TORC1 activity (Fig. 3A). Indeed, in the Sch9_2d3e background Tod6 localization no longer responded to rapamycin (Fig. S3B), contrary to what was seen for Sfp1, which still responded normally (Fig. S3A).

Quantification of the Tod6 N/C ratio during the cell cycle of the Sch9_2d3e strain showed that all features of the wild-type Tod6 N/C ratio during G1, budding and karyokinesis had disappeared, but the peak in G2/M was still present (Fig. 3B; Fig. S3E,M). This observation suggests that Tod6 localization dynamics during G1, budding and karyokinesis are caused by changes in TORC1 activity, whereas the localization of Tod6 in G2/M is not controlled by TORC1.

We next asked whether Tod6 localization during G2/M is controlled by PKA or TORC1-independent Sch9 activity. To investigate this, we monitored the localization pattern of a GFP-tagged Tod6 mutant on which all known Sch9 and PKA phosphorylation sites were mutated to alanine to prevent phosphorylation (Tod6_6A), making this mutant independent of PKA and TORC1 (Huber et al., 2009) (Fig. 3A). Since overexpression of Tod6_6A causes a slow-growth phenotype (Huber et al., 2009), we expressed Tod6_6A from an additional chromosomally integrated copy driven by the endogenous Tod6 promoter, leaving the wild-type protein in place. First, we verified by monitoring TORC1 and PKAas perturbations (via rapamycin, 1-NM-PP1 and CHX) that Tod_6A no longer responded to changes in TORC1 and PKA activity (Fig. S3). We also found that the average N/C ratio of Tod6_6A was higher than in the wild type (Fig. 3C), confirming that unphosphorylated Tod6 accumulates in the nucleus. Finally, the N/C profile of Tod6_6A during the cell cycle was similar to that of the Sch9_2d3e strain (Fig. 3D; Fig. S3).

Taken together, the observations made with the two mutants indicate that the increase in the N/C ratio of wild-type Tod6 during G2/M is not generated by TORC1 or PKA. On the other hand, the lack of wild-type localization features in G1, budding and karyokinesis in these mutants supports the notion that TORC1 and PKA cause the observed wild-type Tod6 localization changes in those phases.

In summary, by comparing our readouts in wild-type and mutant cells we could infer that TORC1 and PKA activity peaks during G1, and decreases as cells approach budding and late mitosis. On the other hand, it is still unclear whether a second activity maximum exists in G2/M, as the Sfp1 data suggest.

### TORC1 and PKA activity oscillations are partially mediated by their nutrient-sensing regulators

If TORC1 and PKA activity oscillates during the cell cycle, which mechanisms could be responsible for these oscillations? We reasoned that perturbation of components involved in the generation of TORC1 and PKA activity oscillations would manifest itself in altered localization patterns of Sfp1 and Tod6. The recent observation of metabolic oscillations (Papagiannakis et al., 2017) that are coupled to the cell cycle and synchronous with the growth rate oscillations prompted us to investigate whether upstream nutrient-sensing regulators of TORC1 and PKA could be implicated in the generation of the observed activity patterns. TORC1 responds to internal metabolic signals generated by carbon and nitrogen sources via two mechanisms: one involving the small GTPases Gtr1 and Gtr2 and the vacuolar EGO complex (Binda et al., 2009; Prouteau et al., 2017), and another involving the vacuolar protein Pib2 (Brito et al., 2019; González and Hall, 2017; Ukai et al., 2018; Varlakhanova et al., 2017), which was recently shown to be a glutamine sensor (Tanigawa et al., 2021). Joint deletion of Gtr1 and Gtr2 with Pib2 is lethal (Ukai et al., 2018), though it is unclear whether the two mechanisms act independently of each other (Ukai et al., 2018; Varlakhanova et al., 2017).

**Fig. 4.**
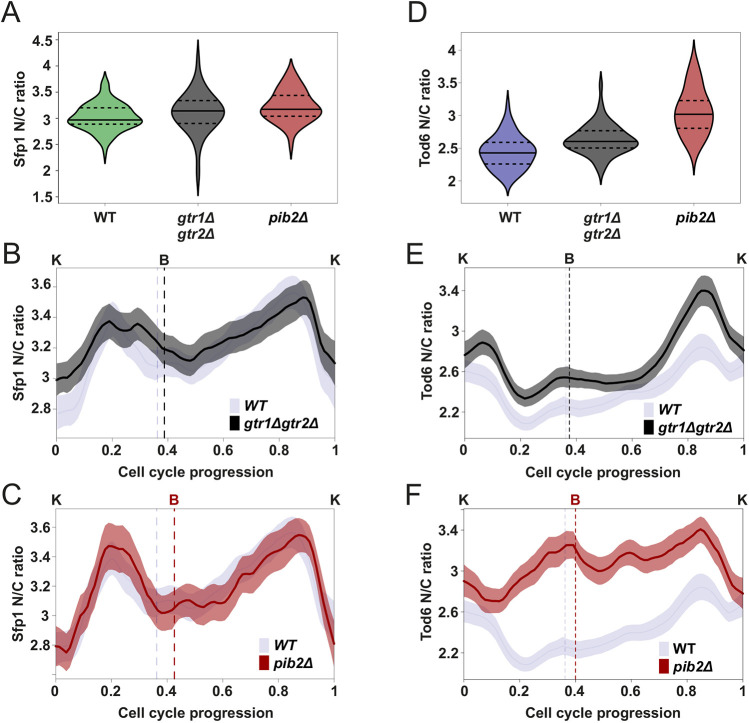
**Pib2 mediates TORC1 activity changes towards Tod6 during the cell cycle.** (A) Sfp1 N/C ratio distributions in the wild-type (WT; *n*=70), and the *gtr1Δgtr2Δ* (*n*=60) and *pib2Δ* (*n*=43) mutants. Median (continuous line) and 25th and 75th percentiles (dashed lines) are displayed. Statistical comparison, two-tailed Mann–Whitney test: *gtr1Δgtr2Δ* to WT, *P*=0.033, *r*=0.21; *pib2Δ* to WT, *P*=3.5*×*10^−3^, *r*=0.4. Even though the second *P*-value is small, the observed effect (difference in medians) is not large. (B) Average Sfp1 N/C ratio dynamics in the *gtr1Δgtr2Δ* background (*n*=122) compared to the wild-type (*n*=149) dynamics (note: wild-type data same as in Fig. 2C, repeated here to facilitate visual comparison). The averages were calculated without normalization of the single-cell data. Cell cycle traces were interpolated and aligned as described in Fig. 2A. Bands denote the 95% confidence interval for the mean. (C) Average Sfp1 N/C ratio dynamics in the *pib2Δ* background (*n*=79) compared to the wild-type (*n*=149) dynamics (note: wild-type data same as in Fig. 2C, repeated here to facilitate visual comparison). Averages were calculated from single-cell traces similarly to in B. (D) Tod6 N/C ratio distributions in the wild-type (*n*=64), and the *gtr1Δgtr2Δ* (*n*=57) and *pib2Δ* (*n*=61) mutants. Median (continuous line) and 25th and 75th percentiles (dashed lines) are displayed. Statistical comparison, two-tailed Mann–Whitney test: *gtr1Δgtr2Δ* to WT *P*=6.6×10^−6^, *r*=0.47; *pib2Δ* to WT *P*=6.5×10^−17^, *r*=0.86. Effect sizes were larger, especially in *pib2Δ* cells. (E) Average Tod6 N/C ratio dynamics in the *gtr1Δgtr2Δ* background (*n*=136) compared to the wild-type (*n*=161) dynamics (note: wild-type data same as in Fig. 2E, repeated here to facilitate visual comparison). Averages were calculated from single-cell traces similarly to in B. It is interesting to observe that the stronger G2 peak in the N/C dynamics of the mutant strain is a consequence of better synchronization in the single-cell G2 localization pulses of Tod6 (Fig. S4). (F) Average Tod6 N/C ratio dynamics in the *pib2Δ* background (*n*=113) compared to the wild-type (*n*=161) dynamics (note: wild-type data same as in Fig. 2C, repeated here to facilitate visual comparison). Averages were calculated from single-cell traces similarly to in B.

To explore the potential contribution of Gtr1, Gtr2 and Pib2 to the TORC1 activity fluctuations during the cell cycle, we generated *gtr1Δgtr2Δ* and *pib2Δ* strains and monitored the localization dynamics of Sfp1 and Tod6 during their cell cycle. We did not observe any significant changes in the average N/C ratio and localization dynamics of Sfp1 in both *gtr1Δgtr2Δ* and *pib2Δ* cells (Fig. 4A–C; Fig. S4). Similarly, although the average N/C ratio of Tod6 was slightly increased in *gtr1Δgtr2Δ* cells, its localization dynamics were similar to that seen in wild type, implying that TORC1 activity was not greatly affected in this mutant (Fig. 4D,E; Fig. S4). On the other hand, deletion of Pib2 caused a marked increase in the average Tod6 N/C ratio, indicating a reduction in TORC1 activity (Fig. 4D). Pib2 deletion also caused significant changes in the Tod6 localization pattern throughout the cell cycle (Fig. 4F; Fig. S4) suggesting that Pib2 is implicated in the generation of the Tod6 localization pattern via the TORC1-Sch9 signaling branch.

To investigate whether Gtr1 and Gtr2 can compensate for the absence of Pib2, we mutated Gtr1 and Gtr2 by inserting point mutations (Gtr1_Q65 L and Gtr2_S23 L) that lock the Gtr proteins into their TORC1-activating configuration (Gtr1-GTP and Gtr2-GDP) (Binda et al., 2009; Nakashima et al., 1999). However, the Tod6 localization pattern in *pib2Δ*_Gtr1_Q65L_Gtr2_S23 L cells remained similar to that in the *pib2Δ* background (Fig. S4), and the average Sfp1 N/C ratio again remained unaffected (Fig. S4).

We next asked whether the differences in Tod6 localization between wild-type, *gtr1Δgtr2Δ* and *pib2Δ* cells were also connected with changes in cell growth. To this end, we determined by microscopy the volume distribution of a cell population at two critical cell cycle points (budding and karyokinesis), and the lengths of cell cycle phase durations in these two strains. As shown in Fig. S5, *gtr1Δgtr2Δ* cells are indistinguishable from wild type with respect to all these metrics. On the other hand, *pib2Δ* mother cells were significantly smaller than the wild type at budding and karyokinesis, and spent more time in both G1 and S/G2/M, resulting in a small increase in total cell cycle duration. As expected from the Sfp1 and Tod6 N/C ratio profiles (Fig. S4), mutation of Gtr1 and Gtr2 to their constitutively active form in a *pib2Δ* background did not fully reverse these changes, as it restored the wild-type cell cycle phase durations, but not the cell volumes.

Altogether, mutations of upstream TORC1 regulators demonstrate that Pib2 is involved in the generation of the TORC1 activity fluctuations during the cell cycle while affecting cell size and the durations of both G1 and S/G2/M. However, this cannot be said for Gtr1 and Gtr2. It is still possible that Gtr1 and Gtr2 participate in the regulation of TORC1 during the cell cycle, but that their contribution is not significant under the high glucose conditions tested here. Alternatively, the loss of these proteins could somehow be compensated for via feedback regulation, which abounds in the TORC1 network (Eltschinger and Loewith, 2016). In any case, the different responses of our readouts in *pib2Δ* and *gtr1Δgtr2Δ* cells, along with the fact that constitutively active Gtr1 and Gtr2 do not fully reverse the effects of Pib2 deletion, suggest that these two upstream regulators of TORC1 are not entirely interchangeable during unperturbed growth.

Next, we evaluated the effect of upstream PKA regulators. Recently, it has been shown that the Ras2 GTPase couples glycolytic flux to PKA activation (Peeters et al., 2017), whereas both TORC1 and PKA pathways are implicated in intracellular pH homeostasis via Gtr1, Gtr2 and the vacuolar ATPase (Deprez et al., 2018; Oliveira et al., 2015). PKA activity is regulated at several points along the pathway: the kinase subunits Tpk1–Tpk3 are repressed by the binding of the regulatory subunit Bcy1, an interaction that is abrogated by the binding of cAMP to Bcy1 (Santangelo, 2006; Toda et al., 1987). cAMP levels are in turn controlled by Ras2 and the Gpa2 Gα protein, which stimulate the adenylate cyclase Cyr1 (Broach, 1991; Santangelo, 2006).

**Fig. 5.**
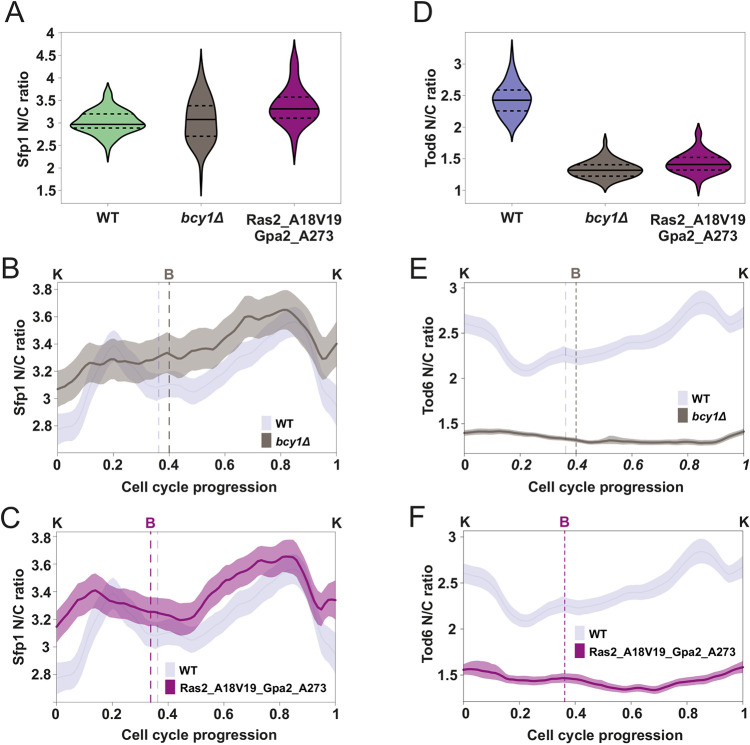
**Bcy1, Ras2 and Gpa2 mediate PKA activity changes towards Sfp1 and Tod6 during the cell cycle.** (A) Sfp1 N/C ratio distributions in the wild-type (WT; *n*=70) and the two mutants with hyperactive PKA (*bcy1Δ*, *n*=62; Ras2_A18V19_Gpa2A273, *n*=69). Median (continuous line) and 25th and 75th percentiles (dashed lines) are displayed. Statistical comparison, two-tailed Mann–Whitney test: *bcy1Δ* to WT, *P*=0.77, *r*=0.03; Ras2_A18V19_Gpa2_A273 to WT *P*=2.1×10^−9,^ *r*=0.59. (B) Average Sfp1 N/C ratio dynamics in the *bcy1Δ* background (*n*=125) compared to wild-type (*n*=149) dynamics (note: wild-type data same as in Fig. 2C, repeated here to facilitate visual comparison). The averages were calculated without normalization of the single-cell data. Cell cycles were interpolated and aligned as described in Fig. 2A. Bands denote the 95% confidence interval for the mean. (C) Average Sfp1 N/C ratio dynamics in the Ras2_A18V19_Gpa2_A273 background (*n*=111) (in which Ras2 and Gpa2 are locked in their active forms) compared to the wild-type (*n*=149) dynamics (note: wild-type data same as in Fig. 2C, repeated here to facilitate visual comparison). Averages were calculated from single-cell data similarly to panel B. (D) Tod6 N/C ratio distributions in the wild-type (*n*=64) and the two mutants with hyperactive PKA (*bcy1Δ*: *n*=59, Ras2_A18V19_Gpa2A273: *n*=46). Median (continuous line) and 25th and 75th percentiles (dashed lines) are also displayed. Statistical comparison, two-tailed Mann–Whitney test: *bcy1Δ* to WT, *P*=1.2×10^−21^, *r*=1; Ras2_A18V19_Gpa2_A273 to WT, *P*=1.4×10^−17^, *r*=0.95. (E) Average Tod6 N/C ratio dynamics in the *bcy1Δ* background (*n*=131) compared to the wild-type (*n*=161) dynamics (note: wild-type data same as in Fig. 2E, repeated here to facilitate visual comparison). Averages were calculated from single-cell data similarly to in B. (F) Average Tod6 N/C ratio dynamics in the Ras2_A18V19_Gpa2_A273 background (*n*=100) compared to the wild-type (*n*=161) dynamics (note: wild-type data same as in Fig. 2E, repeated here to facilitate visual comparison). Averages were calculated from single-cell data similarly to in B.

To determine the contribution of upstream PKA regulators to the observed activity fluctuations, we used the hyperactive PKA mutants *bcy1Δ* and Ras2_A18V19_Gpa2_A273 (the constitutively active forms of Ras2 and Gpa2). Deletion of Bcy1 results in (constitutively) hyperactive Tpk1–Tpk3, and the constitutively active forms of Ras2 and Gpa2, also hyperactivate the PKA pathway (Jorgensen et al., 2004; Kraakman et al., 1999; Schmelzle et al., 2004). In both strains, we did not observe major changes in the average Sfp1 N/C ratio, but the amplitude of the N/C oscillations was reduced, especially in *bcy1Δ* (Fig. 5A–C; Fig. S5). In particular, the minima around budding and karyokinesis (corresponding to low-activity periods of TORC1 and PKA in wild type) were much shallower, consistent with the fact that PKA is constitutively (hyper)active in these strains. On the other hand, the nuclear localization of Tod6 showed a dramatic decrease, whereas the N/C oscillations were greatly suppressed and displayed an altered pattern (Fig. 5D–F; Fig. S5). These observations are again consistent with a hyperactive PKA throughout the cell cycle.

Turning to the cell growth metrics considered above, we did not observe changes in the duration of G1 and S/G2/M of *bcy1Δ* cells, but these cells were much larger than wild type (Fig. S5). The Ras2-Gpa2 mutant strain showed a behavior similar to *bcy1Δ* (Fig. S5).

In summary, perturbations of key upstream regulators of TORC1 and PKA generated changes in the cell cycle patterns of our readouts, consistent with the notion that the upstream, nutrient-sensing nodes (Pib2 in the case of TORC1, and Bcy1, Ras2 and Gpa2 in the case of PKA) are part of the mechanism that generates the activity fluctuations in TORC1 and PKA during an unperturbed cell cycle.

### The synthesis rate of ribosomal proteins Rpl13a and Rpl26a reflects TORC1 and PKA activity changes during the cell cycle

Having observed that TORC1 and PKA activity towards readouts connected with ribosome biogenesis oscillates in synchrony with the cell cycle, we investigated whether this activity pattern is reflected in ribosome biogenesis dynamics. To explore how ribosome biogenesis proceeds during the cell cycle, we used the synthesis of ribosomal proteins (RPs) as a proxy. Previous attempts to quantify yeast RP synthesis were based on bulk measurements of cultures synchronized via centrifugal elutriation (Elliott et al., 1979; Shulman et al., 1973). However, the limitations of bulk synchronization and analysis methods are particularly relevant when studying the cell cycle dynamics of mother cells growing in rich nutrients, where cell cycle duration is the shortest.

To overcome these limitations, we turned again to time-lapse fluorescence microscopy. To measure RP synthesis dynamics in growing single cells, we tagged two ribosomal proteins, Rpl13a and Rpl26a with sfGFP (tagging did not affect cell growth; Fig. S6). TORC1 has been reported to control Rpl13a expression (Marion et al., 2004), and Rpl26a promoter activity has been used as a TORC1 reporter in liquid cell cultures (Kessi-Pérez et al., 2019).

We measured the total abundance of Rpl13a–GFP and Rpl26a–GFP in single cells using the same cell cycle indicators and alignment procedure as above. For each cell cycle, we calculated the total GFP abundance over time, smoothed the resulting time series, and estimated the GFP synthesis rate at each point in time via differentiation and correction for GFP maturation (see Materials and Methods). Using budding and karyokinesis, we then aligned the single-cell traces from cycles of different lengths on a common (relative) time axis denoting cell cycle progression (see Materials and Methods, and Fig. 2A).

**Fig. 6.**
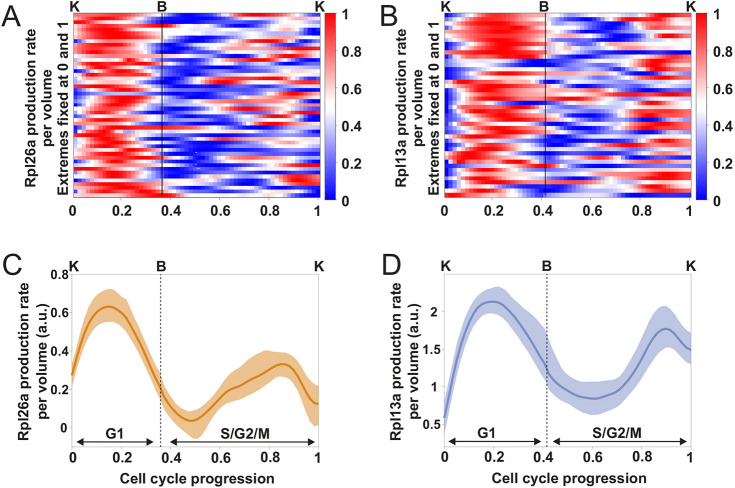
**The synthesis rate of the ribosomal proteins Rpl13a and Rpl2a oscillates in synchrony with the cell cycle.** (A,B) Heatmaps of single-cell estimates of Rpl26a and Rpl13a synthesis rate per unit of volume (*n*=44 cell cycles for Rpl13a, *n*=49 cell cycles for Rpl26a). Time series of individual cell cycles were interpolated and aligned as described in Fig. 2A. For each cell cycle, the time series of the RP synthesis rate was normalized by assigning its maximum to 1 and its minimum to 0, to facilitate the identification of peaks and troughs. Cells were grown in glucose minimal medium over several cell cycles and imaged with a fast frame rate (3 min). (C,D) Average Rpl26a and Rpl13a synthesis rate per unit volume for the cells shown in A and B. The averages were calculated without normalization of the single-cell data. The bands denote the 95% confidence interval for the mean. a.u., arbitrary units.

We found that the synthesis rate of Rpl13a–GFP and Rpl26a–GFP per unit of volume presents two maxima (in G1 and in G2/M) and two minima (after budding and during mitosis) (Fig. 6A,B), indicating that the synthesis rate of Rpl13a and Rpl26a is strongly regulated during the cell cycle (Fig. 6C,D). The observed RP synthesis dynamics are in good agreement with the inferred TORC1 and PKA activity pattern around G1. To further investigate this connection, we quantified the Rpl13a–GFP synthesis rate in mother cells treated with a sublethal dose of rapamycin (see Materials and Methods). As expected, treated cells had a considerably longer G1 phase compared to the wild type (average G1 duration: wild type, 41 min; rapamycin, 160 min). Consistent with TORC1 inhibition, the Rpl13a–GFP synthesis rate showed a lower peak during G1, in agreement with the correspondingly lower peak of Sfp1 localization (Fig. S6). Given that the TORC1 and PKA pathways are the main regulators of ribosome synthesis (Jorgensen et al., 2004; Lippman and Broach, 2009; Powers and Walter, 1999; Zurita-Martinez and Cardenas, 2005) the determined RP synthesis rate further supports our finding that the activity of these signaling pathways oscillates during the cell cycle.

## DISCUSSION

By using time-lapse fluorescence microscopy and meticulous processing of single-cell data, we found evidence that the activity of TORC1 and PKA towards ribosome biogenesis oscillates in synchrony with the cell cycle. Using the intracellular localization of Sfp1 and Tod6 as a proxy for TORC1 and PKA activity, we observed coordinated changes in our two readouts in unperturbed cell cycles of individual mother cells and found that TORC1 and PKA activity shows a maximum during G1 and two minima – one around budding and another in late M/early G1. Meanwhile, it remains unclear what the pattern of TORC1 and PKA activity is during G2. Nutrient-sensing mechanisms upstream of TORC1 and PKA are involved in the regulation of TORC1 and PKA during the cell cycle, suggesting that these signaling pathways respond in part to oscillatory internal metabolic signals during the cell cycle (Amponsah et al., 2021; Baumgartner et al., 2018; Papagiannakis et al., 2017). Finally, the synthesis rate of two ribosomal proteins displayed oscillations in good agreement with the inferred TORC1 and PKA oscillations, further supporting the notion that TORC1 and PKA activity oscillates during the cell cycle.

Through the characterization of Sfp1 and Tod6 localization dynamics via chemical perturbation experiments, we obtained new insights into the regulation of these proteins and opened up directions for future work. For instance, Sfp1 localization seems to be directly regulated by PKA, given the fast response of Sfp1 localization to the 1NM-PP1 perturbation. This observation is further supported by the fact that an Sfp1 mutant with all known TORC1 sites mutated to alanine (Sfp1-1; Lempiäinen et al., 2009) still responded to our chemical perturbations (Fig. S3), confirming earlier observations that this mutant retains some wild-type functionality, and suggesting that additional phosphorylation sites exist on the protein (Lempiäinen et al., 2009) or that Sfp1 localization is additionally regulated through the binding of other proteins (e.g. Mrs6) that are targets of signaling themselves (Lempiäinen et al., 2009; Singh and Tyers, 2009). We also demonstrated a connection between Tod6 phosphorylation and localization, showing that the latter responds to changes in TORC1 and PKA activity. Understanding the mechanisms by which TORC1 and PKA regulate Sfp1 and Tod6 localization will be instrumental for the development of biosensors specific to TORC1 or PKA in the future.

The inferred peak in TORC1 and PKA activity during G1 is in agreement with their role in regulating G1 progression and the G1/S transition (Barbet et al., 1996; Hall et al., 1998; Mizunuma et al., 2013; Polymenis and Schmidt, 1997). Our observation that the Sfp1 peak N/C ratio in G1 appears on average 5–10 min after the peak of Whi5 nuclear localization (i.e. when the nuclear concentration of Whi5 has already started declining), warrants further investigation to understand to what degree the nuclear exit of Whi5 and peak of Sfp1 are causally connected. Moreover, we recently showed that transition through the G1 checkpoint (also known as ‘Start’) is triggered by a pulse in protein synthesis during G1, which, in turn, causes a pulse in the concentration of the unstable G1 cyclin Cln3 (Litsios et al., 2019). Both TORC1 and PKA have been reported to promote traversal through Start by adjusting global protein synthesis (and thus Cln3 levels) in response to nutrients (Barbet et al., 1996; Hall et al., 1998; Mizunuma et al., 2013; Polymenis and Schmidt, 1997). Our results might therefore provide the cause of the observed pulses in protein synthesis and Cln3 concentration during G1, something that also needs to be further explored in the future. The decrease in TORC1 and PKA activity around the moment of budding corroborates previous observations showing that polarized growth (induced by mating pheromone treatment or in cell cycle mutants) decreases TORC1 activity (Goranov et al., 2013). Although we could not resolve TORC1 and PKA activity dynamics in G2/M, the observations of the mutant strains (Fig. S5), as well as the second pulse in RP expression in G2/M indicate that these pathways are active also during G2. Finally, although the budding yeast and mammalian mitosis have several differences, it is interesting to note that the decrease in TORC1 and PKA activity around karyokinesis strongly resembles the growth dynamics observed in mammalian cells, where mass accumulation and protein synthesis persist through prophase, slow down as cells approach the metaphase-to-anaphase transition, and recover during late cytokinesis (Miettinen et al., 2019).

Our investigation of TORC1 and PKA activity dynamics in response to mutations and deletions in their nutrient-sensing regulators showed that Pib2 deletion causes a significant change in Tod6 localization and has a significant effect on volume during the cell cycle. The fact that the Sfp1 localization pattern was not altered significantly in this mutant suggests that Pib2 has a larger effect on the Sch9 branch of TORC1 signaling compared to Sfp1 phosphorylation. The presumed decrease in Tod6 phosphorylation in *pib2Δ* cells (inferred from the increase in the average Tod6 N/C ratio) would be consistent with the fact that Sch9 phosphorylation (and, hence, activity) shows a decrease in *pib2Δ* cells compared to wild type (Ukai et al., 2018). Although both Pib2 and Gtr1 and Gtr2 were known to modulate TORC1 activity in response to nitrogen source shifts and noxious stressors, it was not clear whether they played a role in normal cell cycle progression. Our results suggest that Pib2, a recently discovered sensor of intracellular glutamine in budding yeast (Tanigawa et al., 2021), could relay internal metabolic signals that also modulate TORC1 activity during the cell cycle. Upstream regulators of PKA, such as Bcy1, and Ras2 and Gpa2, are also involved in the modulation of PKA activity, as evidenced by the changes induced on the localization of both Sfp1 and Tod6 upon Bcy1 deletion and the locking of Ras2 and Gpa2. Notably, Bcy1 binding to Tpk1–Tpk3 is regulated by cyclic AMP, a central signaling metabolite, whereas further upstream, Ras2 activity has been shown to respond to changes in glycolytic flux (Peeters et al., 2017). It remains to be seen precisely which metabolic inputs are in fact responsible for the observed TORC1 and PKA activity changes during each cell cycle phase.

Overall, our results demonstrate that the activity of two central pathways controlling cell growth is temporally organized and tightly connected with cell cycle progression. Our observation that TORC1 and PKA activity fluctuates in synchrony with the cell cycle is in line with a recent report showing that metabolic precursors are synthesized at different rates during the cell cycle (e.g. amino acid synthesis increases in G1 and G2/M with a minimum in S) (Campbell et al., 2020) and the observation that metabolism operates as an autonomous oscillator that is coupled to the cell cycle (Papagiannakis et al., 2017). On the other hand, it becomes increasingly apparent that growth-controlling signaling pathways are in direct, bidirectional communication with the cell cycle machinery (Moreno-Torres et al., 2017, 2015; Odle et al., 2020; Romero-Pozuelo et al., 2020; Talarek et al., 2017). It will therefore be instructive to investigate further whether the activity of TORC1 and PKA towards catabolic processes such as autophagy is also coordinated with the cell cycle, as recent evidence suggests (Li et al., 2020; Odle et al., 2020; Yamasaki et al., 2020). Untangling the complex interactions of these fundamental intracellular processes will provide a much deeper understanding of how proliferating cells coordinate growth with division.

## MATERIALS AND METHODS

### Yeast strains

All strains were constructed on the S288C-derived prototrophic YSBN6 background (Canelas et al., 2010). Integration of fluorescent reporters was carried out using homologous recombination. Fragments containing the fluorescent protein, the resistance cassette and the appropriate flanking sequences were amplified from plasmids built via Gibson assembly. Replacements of Bcy1 and Pib2 with the resistance cassette were constructed the same way. The sequence for the mutant Tod6_6A(S280A, S298A, S308A, S318A, S333A, S346A) was taken from plasmid pAH268 (Huber et al., 2009). The sequence for the mutant Sfp1-1(S39A, S170A, S181A, S183A, T227A, T228A, T446A) was taken from plasmid pHL150 (Lempiäinen et al., 2009). The sequence for the mutant Sch9_2D3E(T723D, S726D, T737E, S758E, S765E) was taken from plasmid pJU841 (Urban et al., 2007). Strains containing deletion of Gtr1 or Gtr2 and mutation of Tpk1(M164G), Tpk2(M147G), Tpk3(M165G), Gtr1(Q65L), Gtr2(S23L), Ras2(A18_V19) or Gpa2(A273) were constructed via CRISPR-Cas9 using the MoClo Yeast Toolkit (Lee et al., 2015). Target sequences used for CRISPR-Cas9 mutagenesis and deletions can be found in Table S1. All strains were verified by PCR and sequencing.

### Yeast growth media and cultivation

Cells were grown in minimal medium (Verduyn et al., 1992) supplemented with 2% glucose (Sigma-Aldrich). All batch cultures were carried out at 30°C with shaking at 300 rpm, and exponentially growing cells were used for all microscopy experiments.

To test the effect of continuous TORC1 inhibition on ribosomal protein synthesis, rapamycin (Sigma-Aldrich) was added to wild-type cells growing exponentially in minimal medium containing 2% glucose to a final concentration of 200 ng/ml. Cells were then left to grow for 2 h before being placed under the microscope. For microscopy, an agar pad (minimal medium plus 2% glucose and 1% agarose) with aforementioned rapamycin concentration was prepared. The agar pad was placed on a glass Petri dish given that rapamycin tends to adsorb on plastic surfaces. Rapamycin was added after letting the medium with melted agarose cool down in order to prevent degradation of the drug.

### Rapamycin preparation for acute treatment experiments involving Sfp1

Treatment of Sfp1–pHtdGFP cells with DMSO alone produced strong Sfp1 delocalization with a timing and magnitude that were comparable to that of rapamycin diluted in DMSO. For this reason, we used a protocol that does not rely on DMSO for the acute treatment of Sfp1–pHtdGFP cells with rapamycin.

A concentrated stock of rapamycin (30 µg/ml) was prepared by dissolving the rapamycin powder in pure methanol. Afterwards, the stock solution was aliquoted in sterile 1.5 ml Eppendorf tubes and stored at −80°C after methanol evaporation. Prior to the experiment, an aliquot was taken from the freezer and the dried rapamycin was resuspended in prewarmed minimal medium (with 2% glucose) with repeated pipetting for optimal mixing. For acute inhibition of TORC1, the medium plus rapamycin solution (2 μl) was directly added to the well plate at a final concentration of 200 ng/ml.

### Microscopy

All microscopy experiments were performed using inverted fluorescence microscopes (Eclipse Ti-E, Nikon Instruments). Temperature was kept constant at 30°C using a microscope incubator (Life Imaging Services). For all the experiments, a 100× Nikon S Fluor (NA=1.30) objective was used. Images were recorded using iXon Ultra 897 DU-897-U-CD0-#EX cameras (Andor Technology). Fluorescence measurements were performed using an LED-based excitation system (pE2; CoolLED Limited and Lumencor, AURA). For GFP measurements, cells were excited at 470 nm (excitation filter, 450–490 nm; dichroic, 495 nm; emission filter, 500–550 nm). For YFP measurements cells were excited at 500 nm (excitation filter, 490–510 nm; dichroic, 515 nm; emission filter, 520–550 nm). For RFP measurements cells were excited at 565 nm (excitation filter, 540–580 nm; dichroic, 590 nm; emission filter, 600–650 nm). During brightfield imaging, a long-pass (600 nm) filter was used. The Nikon Perfect Focus System (PFS) was used to prevent loss of focus.

For imaging in unperturbed conditions, cells were placed under a prewarmed agar pad (minimal medium, 2% glucose, 1% agarose) and imaged for at least 8 consecutive hours. For each experiment, multiple non-overlapping *xy* positions were recorded and for each position brightfield and fluorescent images were recorded. For imaging of Rpl13a–sfGFP and Rpl26a–sfGFP, three brightfield *z*-axis planes images with a 0.5 µm step were recorded for each *xy* position every 3 min; one GFP and one RFP fluorescence image were recorded for each position, every 3 min, at the focal plane corresponding to the intermediate brightfield image. For all the other experiments, one brightfield, GFP and RFP image were recorded for every *xy* position every 5 min.

For experiments involving chemical perturbations using rapamycin, DMSO, MSX, CHX and 1-NM-PP1 (Sigma-Aldrich), exponentially growing cells at an optical density (OD) at 600 nm of 0.1 were incubated for 30 min at 30°C in plastic well plates for inverted fluorescence microscopy (Ibidi) treated with concanavalin A (1 mg/ml; Sigma-Aldrich). The wells were then washed twice with prewarmed minimal medium (plus 2% glucose) and placed under the microscope. Imaging settings were as reported above. For each well, multiple non-overlapping *xy* positions were recorded and for each position, one brightfield, GFP and RFP image were recorded every 5 min. After at least 1 h from the beginning of the imaging, chemicals were added to the wells at the final concentration of 200 ng/ml for rapamycin (diluted in DMSO/medium), 2 mM for MSX (diluted in H_2_O), 25 µg/ml for CHX (diluted in H_2_O) and 500 nM for 1-NM-PP1 (diluted in DMSO).

### Image analysis

For each experiment, the fluorescence channel images were background-corrected using the rolling ball background subtraction plugin in ImageJ. Cell segmentation and tracking were performed using the semi-automatic ImageJ plugin BudJ (Ferrezuelo et al., 2012). For the calculation of the single-cell N/C ratio of Sfp1–pHtdGFP and Tod6–pHtdGFP, the background-corrected images were subsequently analyzed using a custom-made Python script and the segmentation boundaries detected by BudJ (see below). Mother cell budding events were annotated based on the appearance of a dark spot on the cell membrane, and karyokinesis was annotated based on the first frame when the nucleus of the mother cell and the nucleus of the bud are completely detached. For the calculation of the single-cell synthesis rate of Rpl13a–sfGFP and Rpl26a–sfGFP, the intermediate *z*-stack brightfield image was used for segmentation and tracking of mother cells, whereas the brightfield *z*-stack where the bud was best defined was chosen for the segmentation and tracking of each bud. For the calculation of the synthesis rate, the total (mother plus bud) cell volume and the average GFP fluorescence intensity from BudJ were used for subsequent analysis (see below). For all analyses of cell cycle dynamics, we considered only mother cells, that is cells that had already produced at least one bud.

### Measurement of the Sfp1–GFP and Tod6–GFP nuclear-to-cytosolic ratio in single cells

A schematic of the nuclear-to-cytosolic ratio measurement can be found in Fig. S2. For each *xy* position, the segmentation data from BudJ and the corresponding background-corrected images were read in a custom-made Python script. The segmentation information from BudJ was used to generate a mask of the corresponding cell at each time frame. The nucleus of the cell was segmented by applying a simple intensity threshold in the RFP channel within the cell mask. A small and a large nuclear mask were calculated as circles of radii 0.48 µm and 1.44 µm, respectively, centered at the centroid of the segmented nucleus. Mean GFP intensity in the nucleus was measured by applying the small nuclear mask to the GFP channel and measuring the average pixel intensity inside the mask. Mean cytosolic GFP intensity was calculated by subtracting the big nuclear mask from the cell mask and applying the resulting mask to the GFP channel and calculating the average pixel intensity inside the mask. To quantify the average Sfp1, Tod6 and Stb3 N/C ratio in different strains (Figs 3B, 4A,B, 5E,F, and Fig. S1A) we quantified the average N/C ratio of mother cells growing under agar pads between minute 200 and minute 400 from the start of imaging.

To compare and average Sfp1 and Tod6 N/C ratio during cell cycles of different lengths we first split every cell cycle time series into two parts, one from karyokinesis to the following budding event (G1) and the other from the budding event to the next karyokinesis event (S/G2/M). We then linearly interpolated the first part of every cell cycle time series with a fixed number of equidistant points and did the same with the second part. The number of points chosen for the interpolation of the first and the second part of each cell cycle time series were calculated based on the ratio of the average durations of the first part and the second part of the cell cycle. In total, 80 interpolation points were used for each cell cycle. As an example: for an average cell cycle duration of 100 min and average G1 and S/G2/M durations of 40 min and 60 min, respectively, the first part of every cell cycle would be interpolated with 32 points and the second with 48 points. For subsequent plotting, we either aligned or averaged the interpolated points of each cell cycle on a common (relative) time scale with 80 steps ranging from 0 to 1.

Python and MATLAB scripts for N/C ratio quantification, cell cycle trace alignment and protein synthesis rate quantification are available on Github (https://github.com/amiliasargeitis/microscopy_scripts).

### Determination of cell cycle phase durations and volume at cell cycle events

G1 was defined as the time between karyokinesis and the subsequent budding event, whereas S/G2/M was defined as the time between budding and the subsequent karyokinesis event. To determine cell volume at budding, we quantified with BudJ the volume of mother cells at the first frame where budding was detectable. To determine bud volume at karyokinesis, we quantified with BudJ the volumes of buds at the first frame where their nucleus had completely detached from the nucleus of the mother cell.

### Estimation of the Rpl13a and Rpl26a synthesis rate during the cell cycle

To estimate the single-cell synthesis rate of Rpl13a–GFP and Rpl26a–GFP during the cell cycle, we first multiplied the total volume (mother plus bud) with the mean cellular GFP time series of the mother cell given by BudJ for each cell cycle, obtaining a GFP abundance time-series for each cell cycle. We assumed that during bud growth, Rpl13a–GFP and Rpl26a–GFP concentration is the same in the mother cell and in the bud, and therefore calculated the mean GFP time series based on the segmentation of the mother cell. Each GFP abundance time series was then fitted with a smoothing spline in MATLAB to reject artifacts and outliers produced by the segmentation step. To better fit the GFP abundance time series at the beginning and at the end of each cell cycle, each volume and mean GFP time series were extended with measurements from three frames (9 min) before the first karyokinesis and three frames after the second karyokinesis. These extra time points were then removed from any further analysis.

To obtain the maturation-corrected synthesis rate of Rpl13a–GFP and Rpl26a–GFP during the cell cycle, we considered a simple model of protein production and maturation. The immature (i.e. non-fluorescent) protein is synthesized at a (time-varying) rate *K*_p_(*t*) and matures in its fluorescent form with a constant rate *K*_m_. Defining *P*_i_ as the abundance of the immature protein and *P*_m_ as the abundance of the mature protein, we obtain the following equations that describe the accumulation of our fluorescent protein:

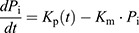


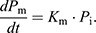
In microscopy experiments, we can actually measure the abundance of the mature protein, i.e. *P*_m_(*t*). To estimate the production rate *K*_p_(*t*) based on *P*_m_(*t*) we use the above equations to arrive at the following:

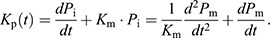
The first derivative of *P*_m_ was estimated from the smoothing spline of the GFP abundance time series. The first derivative time series was then fitted with a second, closely fitting smoothing spline to estimate the second derivative of *P*_m_. In the calculation of *K*_p_(*t*), we considered a maturation half time of 6 min for sfGFP (Khmelinskii et al., 2012), leading to *K*_m_=0.116 min^−1^. We did not consider any degradation of RPs during the cell cycle, as there is no evidence for this type of RP regulation. Plotting and averaging of the Rpl13–GFP and Rpl26–GFP synthesis rates during the cell cycle were done in the same way with the Sfp1 and Tod6 time series. In this case, we fixed the total number of interpolation points at 60.

### Comparing two groups of single-cell time series

#### Problem setting

We start with two sets of noisy single-cell measurements of a cellular readout across two cell populations (e.g. the wild type and a mutant), denoted by 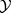 and 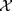. Individual cells are followed over time, and we assume that their readouts are provided at the same time points (this is for example the case when individual time series of individual cells are aligned based on cell cycle events).

We denote by:

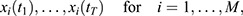
the set of measurements of population 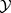, where *i* is the cell index, *M* is the number of quantified cells, and *t*_1_,…, *t*_*T*_ are the measurement time points. Likewise, we let:

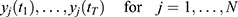
denote the set of measurements of population 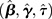, where *j* and *N* denote the cell index and the number of cells, respectively.

Given these data, we want to assess whether the mean trajectories of the two populations differ. Note that carrying out statistical comparisons of measurements at individual time points (e.g. via *t*-tests) is not a statistically sound approach, because the observations of individual cells are correlated in time, and therefore the measurements at different time points are not independent. In order to carry out comparisons, we therefore need to generate population models which take into account the time correlations in the single-cell trajectories. The type of model that we use is described in the following.

#### Notation

All vectors and matrices are denoted with bold font. A vector ***v*** is always assumed to be a column, and ***v^′^*** denotes the transpose of ***v***. Likewise, ***M^′^*** denotes the transpose of a matrix ***M***.

A vector ***v*** written as

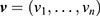
(i.e. a comma-separated list) is assumed to be a column.

#### Modeling

We assume that single-cell time series from population *x* are generated from a model of the form

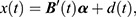
where ***B***(*t*) is a vector of basis functions, ***α*** is a vector of coefficients, and *d*(*t*) is a zero-mean Gaussian process (GP) (Rasmussen and Williams, 2006), i.e. a stochastic process defined so that for every finite set of time indices *t*_1_,…,*t*_*n*_ the vector (*d*(*t*_1_),…,*d*(*t*_*n*_)) follows a zero-mean multivariate Gaussian distribution. The term ***B^′^***(*t*)***α*** is the mean (i.e. the ‘deterministic part’) of *x*(*t*), while *d*(*t*) (the ‘stochastic part’) produces correlated fluctuations around the mean, reflecting the fact that individual cell behaviors are stochastic.

The GP is completely defined by its covariance function *K*(*s*, *t*), which determines the covariance of *d*(*s*) and *d*(*t*) for any pair of time points *s* and *t* (Rasmussen and Williams, 2006). Given that cell populations may display a non-stationary behavior (i.e. variability that changes over time and fluctuations with different length scales), we use a covariance function of the form:

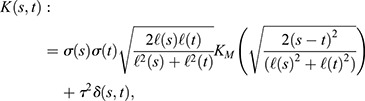
where *σ*(*t*) is the time-varying standard deviation and 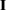 the time-varying length scale of the process. Non-stationary covariance functions such as the one above are generated based on a stationary covariance function *K*_*M*_, as described in Paciorek and Schervish (2003). The choice of *K*_*M*_ determines the features of the generated stochastic process *d*(*t*). For our model, we chose *K*_*M*_ as the Matérn kernel with *ν*=5/2, which produces trajectories that are less ‘smooth’ than those produced by the commonly used squared exponential kernel (Rasmussen and Williams, 2006), and therefore better capture the variability of single-cell data. Note that in the parameterization of the Matérn kernel used here, the argument |*s*−*t*|/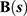 is replaced by the square root shown above due to the use of a time-varying length scale (Paciorek and Schervish, 2003). Finally, the Dirac delta function *δ*(*s*, *t*) scaled by 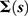 is used to describe measurement noise, i.e. residual (uncorrelated) variability that does not stem from single-cell stochastic behavior.

Altogether, the GP model of single-cell time series includes the unknown vector ***α***, the unknown functions *σ*(*t*) and 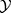 and the parameter 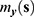. Since *σ*(*t*), 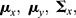 are functions of time, we estimate them using the basis function expansions:

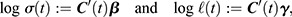
where ***C***(*t*) is a vector of basis functions, ***β***, ***γ*** are vectors of coefficients, and the logarithms ensure that *σ*(*t*), 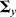 are positive. Both ***B***(*t*) (defined above) and ***C***(*t*) consist of a set of Gaussian kernels with common bandwidth *h* and evenly spaced centers *t*_1_, *t*_1_+*h*, …, *t*_*T*_. We denote the number of kernels in ***B***(*t*) and ***C***(*t*) by *J* and *L*, respectively. The bandwidth for ***B***(*t*) is given by *h*=(*t*_*T*_−*t*_1_)/(*J*−1) and by *h*=(*t*_*T*_−*t*_1_)/(*L*−1) for ***C***(*t*). We used *J*=10 kernels for describing the time-varying mean with sufficient flexibility for accommodating fast changes in the population averages. On the other hand, we used *L*=5 for the time-varying standard deviation and length-scale, which prevents too fast changes in the stochastic behavior of the population that can lead to overfitting. Under these modeling assumptions, the covariance function defined above becomes also a function of ***β***, ***γ*** and *τ*, and will henceforth be denoted by *K*(*s*, *t*; ***β***, ***γ***, *τ*).

Finally, a model for population 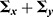 is defined completely analogously. To avoid complicating the notation, we will focus on the 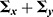 model in the following, but exactly the same operations need to be performed for the 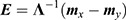 model.

#### Bayesian estimation of GP parameters

The single-cell model defined in the previous section contains parameters ***α***, ***β***, ***γ***, and *τ*. We next provide the specification of prior distributions. We assign a N(0, 10^2^) prior to the components of ***α***, a Γ(1, 1) prior to *τ* and the components of ***β***, and a Γ(0.2(*t*_*T*_−*t*_1_), 1) prior to the components of ***γ***. The prior on ***α*** is sufficiently broad to allow the mean of *x*(*t*) to capture a wide range of responses, while the priors of ***β*** and *τ* are concentrated on a small range of (positive) numbers. This is necessary for preventing too strong oscillations in the standard deviation of the GP (via ***β***), and for adjusting the magnitude of measurement noise to the scale of the measured readouts (via *τ*).

Following the prior specification, we need to estimate the parameterization of the GP part of *x*(*t*). We can do this by finding the Maximum a Posteriori (MAP) estimates of the covariance function parameters ***β***, ***γ***, and *τ*. At this stage, the mean coefficient vector ***α*** is a nuisance parameter that needs to be optimized simultaneously.

Let ***x***_*i*_:=(*x*_*i*_(*t*_1_),…, *x*_*i*_(*t*_*T*_)) denote the vector of measurements for each cell *i*=1,…,*M*. Moreover, denote:

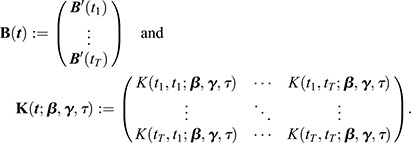
The MAP estimate is found by numerically optimizing the posterior probability:

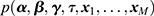


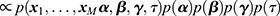


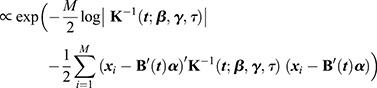


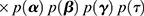
with respect to ***α***, ***β***, ***γ***, and *τ*. Disregarding the optimized value for ***α***, we obtain the MAP estimate 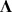.

With this estimation step, the GP part *d*(*t*) of *x*(*t*) is determined. We next turn to the estimation of ***α***, which defines the mean of *x*(*t*). Note that ***α*** was also estimated above, but the large number of degrees of freedom in that optimization problem does not allow us to obtain accurate estimates of ***α*** and its associated uncertainty.

#### Posterior distribution of the mean

The next step is to infer the mean function *m*(*t*):=***B^′^***(*t*)***α*** together with its uncertainty. With the GP part of *x*(*t*) fixed, we can infer ***α*** by solving a Bayesian linear regression problem of the form:

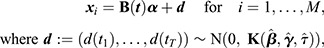
is now a finite-dimensional Gaussian random vector with covariance parameterized as described in the previous step. In this setup, the posterior distribution of ***α*** is a Gaussian (O'Hagan, 1994, p. 246) with mean

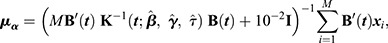
and covariance:

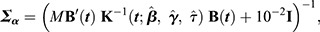
where 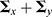 is the *J*×*J* identity matrix and the factor 10^−2^ stems from the prior variance of ***α***.

We can now obtain the posterior distribution of the mean *m*(*t*) at any vector ***s***=(*s*_1_, *s*_2_, …) of time points between *t*_1_ and *t*_*T*_. To do so, we pre-multiply ***α*** by 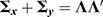, where

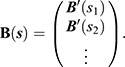
Hence, the posterior distribution of *m*(***s***):=(*m*(*s*_1_), *m*(*s*_2_), …) is also Gaussian with mean

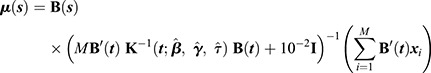
and covariance

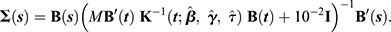
Since *m*(*t*) is defined in terms of *J* basis functions, its posterior covariance matrix 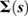 will not be of full rank when ***s*** contains more than *J* points. For this reason, we take ***s*** to be a sparse grid of *J* evenly spaced time points on [*t*_1_, *t*_*T*_], i.e.:

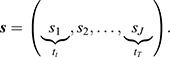
Repeating this procedure for population 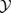, we are left with two posterior mean distributions, one for each cell population. The next subsection details the comparison of these distributions, providing an effect size of their difference and a significance that may be used for hypothesis testing.

#### Inference

From the previous step, we obtained posterior distributions of the posterior means *m*_***x***_(***s***), 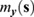 of both populations on the sparse grid ***s*** defined above, where:

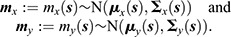
In the remainder, we drop the time arguments of 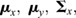 and 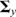 for the sake of readability**.** Since the two populations are independent, it follows that the difference ***m***_*x*_−***m***_*y*_ is normally distributed with mean ***μ***_***x***_−***μ***_***y***_ and covariance matrix 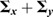. To assess whether ***m***_*x*_−***m***_*y*_ is significantly different from the zero vector, we adopt the idea of Benavoli and Mangili (2015) and inspect

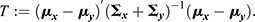
This quantity follows a chi-squared distribution *χ*^2^(*ν*), where *ν* is the number of positive eigenvalues of 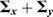. We can then calculate the largest tail probability ε for which the 1−ε credible region of ***m***_*x*_−***m***_*y*_ contains the zero vector or, equivalently, *T* is smaller than the (1−ε)–quantile of the *χ*^2^(*ν*) distribution. A value of ε close to one implies that ***m***_*x*_−***m***_*y*_ is quite likely to be zero, and therefore the two cell populations do not differ from each other. Conversely, a value of ε closer to zero implies that ***m***_*x*_−***m***_*y*_ is most likely non-zero, and therefore the two populations differ. Therefore, ε expresses how significant the difference between the two posterior means is. Note that, since ***m***_*x*_ and ***m***_*y*_ are high-dimensional vectors, ε can be small (around 10^−3^ to 10^−6^) even if the two means only show minor differences.

Due to the fact that ε takes into account the whole vectors of posterior means (i.e. all measured time points together), it can only answer the question of whether the two cell populations differ or not. To break down the differences across time points, we can decompose *T* as:

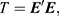
where 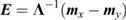 and 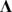 is a Cholesky factor of 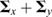 (that is, 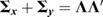). Plotting ***E*** can provide visual guidance on which points of the sparse grid ***s*** produce the strongest deviations between the two population averages.

An example application of our modeling and inference approach, along with MATLAB code to perform the calculations described above (for reproducing the results of Table S2 and Figs S3, S4 and S5) is provided at Github (https://github.com/yulanvanoppen/GPcompare).

## Supplementary Material

10.1242/joces.260378_sup1 Supplementary information
