# Supplementary material for: TORC1 and PKA activity towards ribosome biogenesis oscillates in synchrony with the budding yeast cell cycle
Source: J Cell Sci. 2022 Sep 28;135(18):jcs260378. doi: 10.1242/jcs.260378 (PMC9658999; doi:10.1242/jcs.260378)
Supplement: Supplementary information [file joces-135-260378-s1.pdf]

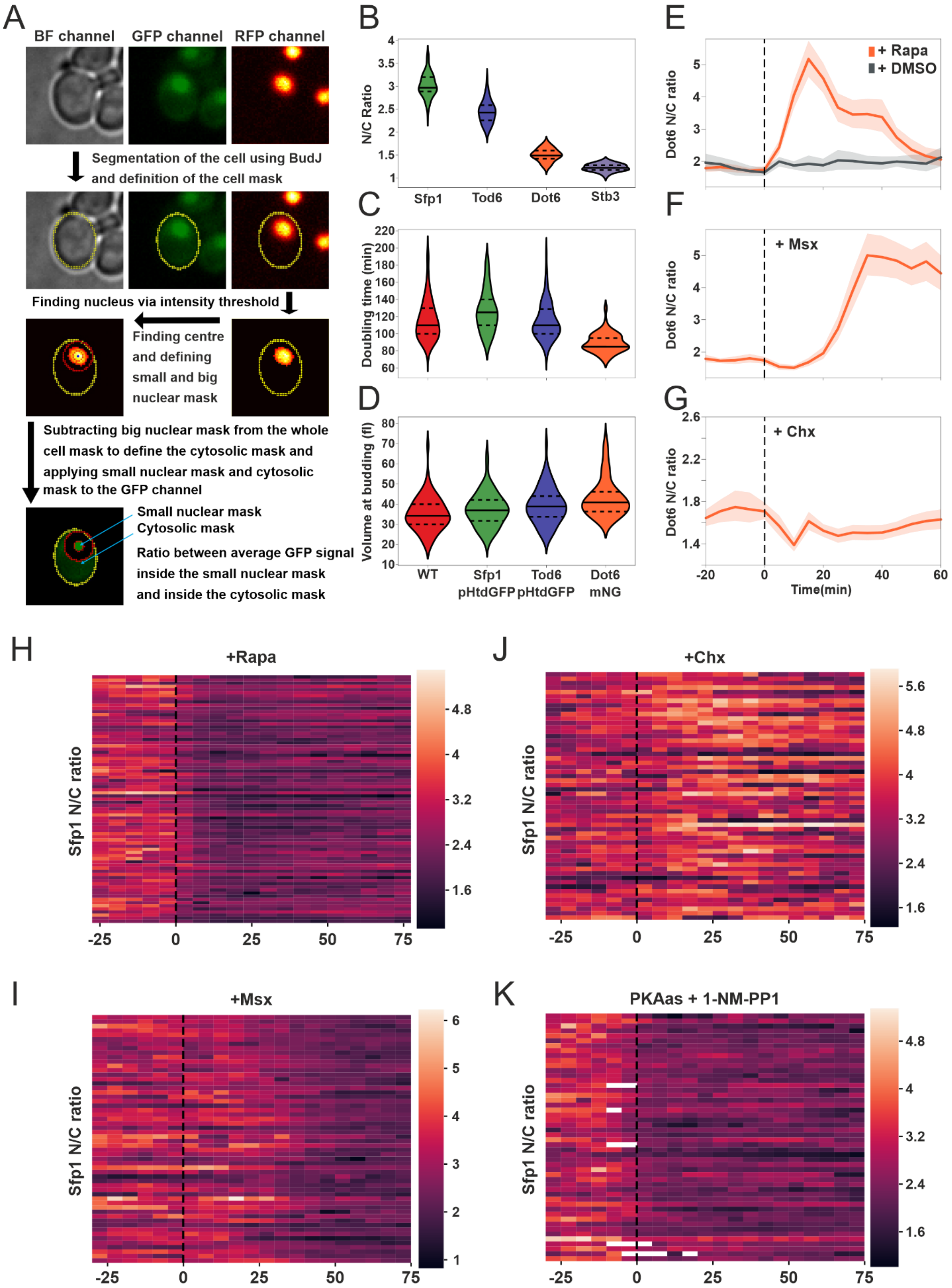
A
BF channel
GFP channel
RFP channel
Segmentation of the cell using BudJ and definition of the cell mask
Finding nucleus via intensity threshold
Finding centre and defining small and big nuclear mask
Subtracting big nuclear mask from the whole cell mask to define the cytosolic mask and applying small nuclear mask and cytosolic mask to the GFP channel
Small nuclear mask
Cytosolic mask
Ratio between average GFP signal inside the small nuclear mask and inside the cytosolic mask
B
N/C Ratio
Sfp1
Tod6
Dot6
Stb3
C
Doubling time (min)
D
Volume at budding (fl)
WT
Sfp1 pHtdGFP
Tod6 pHtdGFP
Dot6 mNG
E
+ Rapa
+ DMSO
Dot6 N/C ratio
F
+ Msx
G
+ Chx
Time(min)
H
+Rapa
Sfp1 N/C ratio
J
+Chx
I
+Msx
K
PKAas + 1-NM-PP1

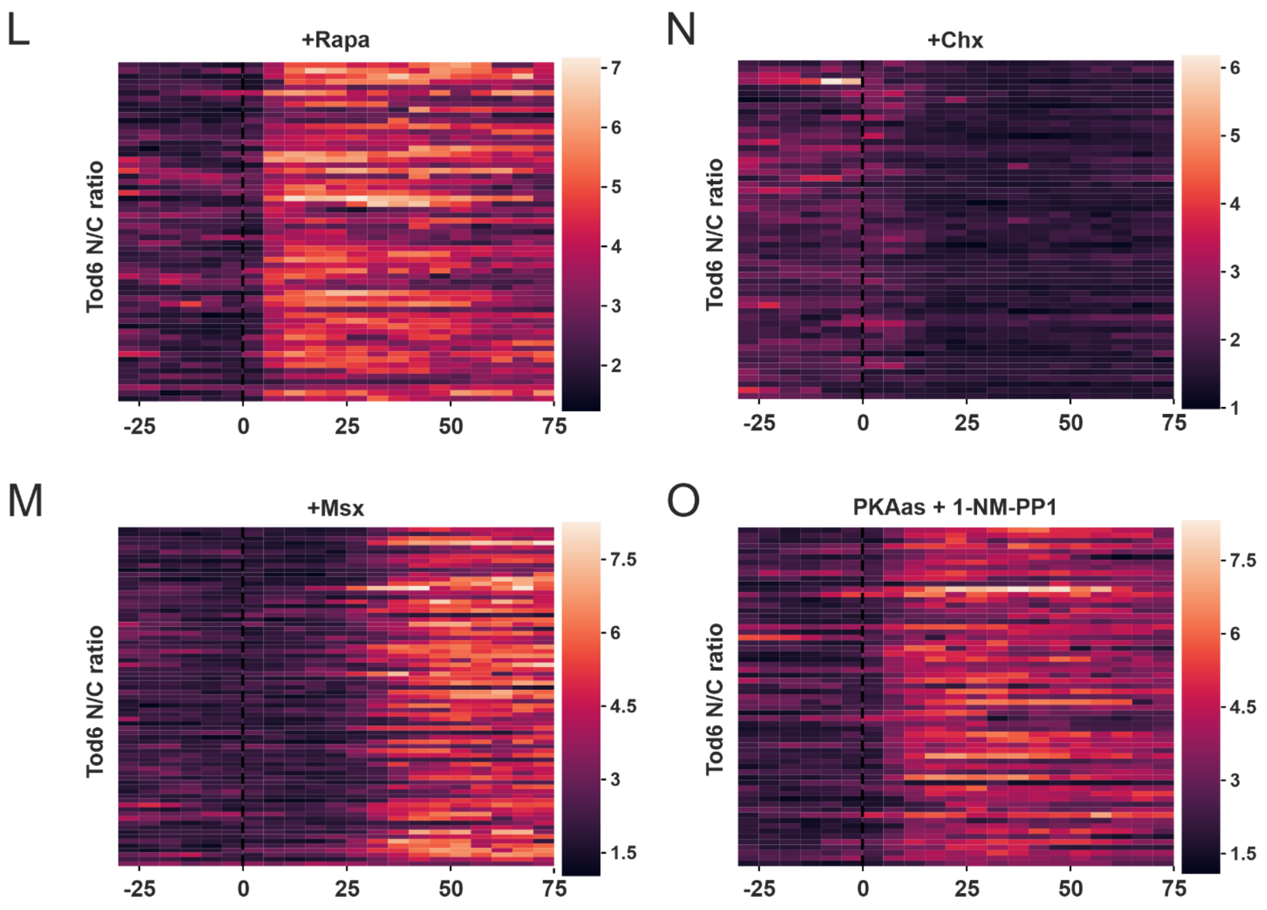

Fig. S1. Related to Figure.1

A. Schematic representation of the pipeline for calculating the Sfp1 and Tod6 N/C ratio in a single cell. **B.** Distributions of single-cell N/C ratios for Sfp1-pHtdGFP (n=70), Tod6-pHtdGFP (n=64), Dot6 (n=52) and Stb3-pHtdGFP (n=53). Median (continuous line) and 25th and 75th percentiles (dashed lines) are also displayed. **C.** Doubling time (karyokinesis-to-karyokinesis) distributions for single mother cells of the wild type (n = 100), Sfp1-pHtdGFP (n = 72), Tod6-pHtdGFP (n = 94) and Dot6-mNG (n = 95) backgrounds. Median (continuous line) and 25th and 75th percentiles (dashed lines) are also displayed. Statistical comparison, two-tailed Mann-Whitney test: Sfp1-WT p-value = 0.002, effect size (rank-biserial correlation) r = 0.26, Tod6-WT p-value = 0.9, r = 0.004, Dot6-WT p-value = $1.1\cdot10^{-19}$, r = 0.75 **D.** Distributions of single-cell volumes at budding for mother cells of the wild type (n = 68), Sfp1-pHtdGFP (n = 71), Tod6-pHtdGFP (n = 77) and Dot6-mNG (n = 93) backgrounds. Median (continuous line) and 25th and 75th percentiles (dashed lines) are also displayed. Statistical comparison, Mann-Whitney test: Sfp1-WT p-value = 0.088, r = 0.16, Tod6-WT p-value = 0.004, r = 0.27, Dot6-WT p-value = $4.5\cdot10^{-8}$, r = 0.5. **E**. N/C ratio dynamics of Dot6 in response to rapamycin (200ng/ml final) (n=44) and its vehicle DMSO (n=16). Rapamycin and DMSO were added at time t=0. The bands denote the 95% confidence interval for the mean. **F.** N/C ratio dynamics of Dot6 in response to methionine sulfoximine (MSX) (2mM final) (n=50). MSX was added at time t=0. The bands denote the 95% confidence interval for the mean. **G.** N/C ratio dynamics of Dot6 in response to cycloheximide (CHX) (25µg/ml final) (n=58). CHX was added at time t=0. The bands denote the 95% confidence interval for the mean. **H-K.** Heatmap of

Journal of Cell Science • Supplementary information

Sfp1-pHtdGFP N/C ratio in individual cells after treatment with rapamycin (n=60), MSX (n=56), CHX (n=56) and 1-NM-PP1 (diluted in DMSO and applied to PKAas cells) (n=50). Experimental details are described and average trajectories are shown in Fig1C-F. **L-O.** Heatmap of Tod6-pHtdGFP N/C ratio in individual cells after treatment with rapamycin (diluted in DMSO) (n=61), MSX (n=75), CHX (n=56) and 1-NM-PP1 (diluted in DMSO and applied to in PKAas cells) (n=63). Experimental details are described and average trajectories are shown in Fig1G-J.

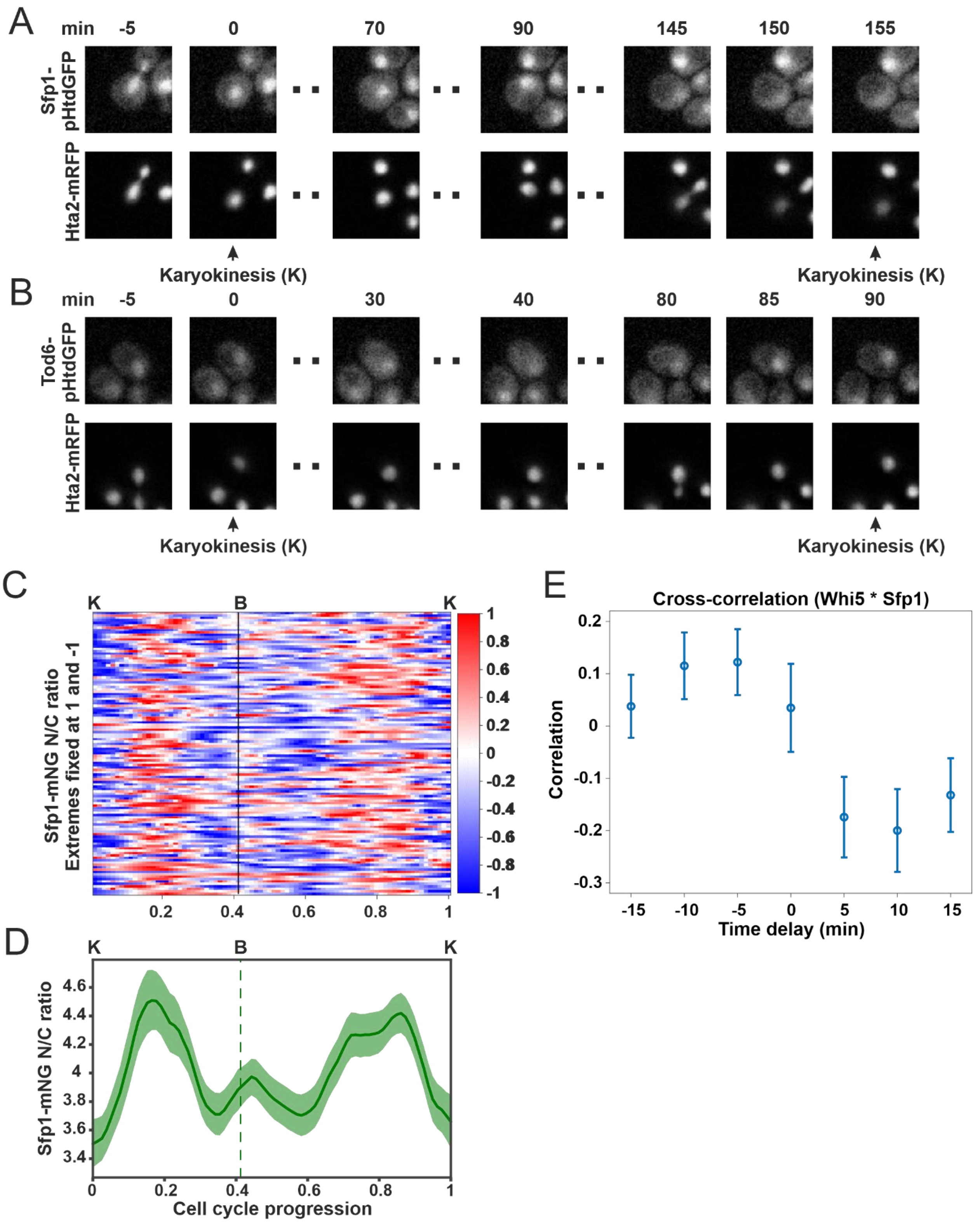

Journal of Cell Science • Supplementary information

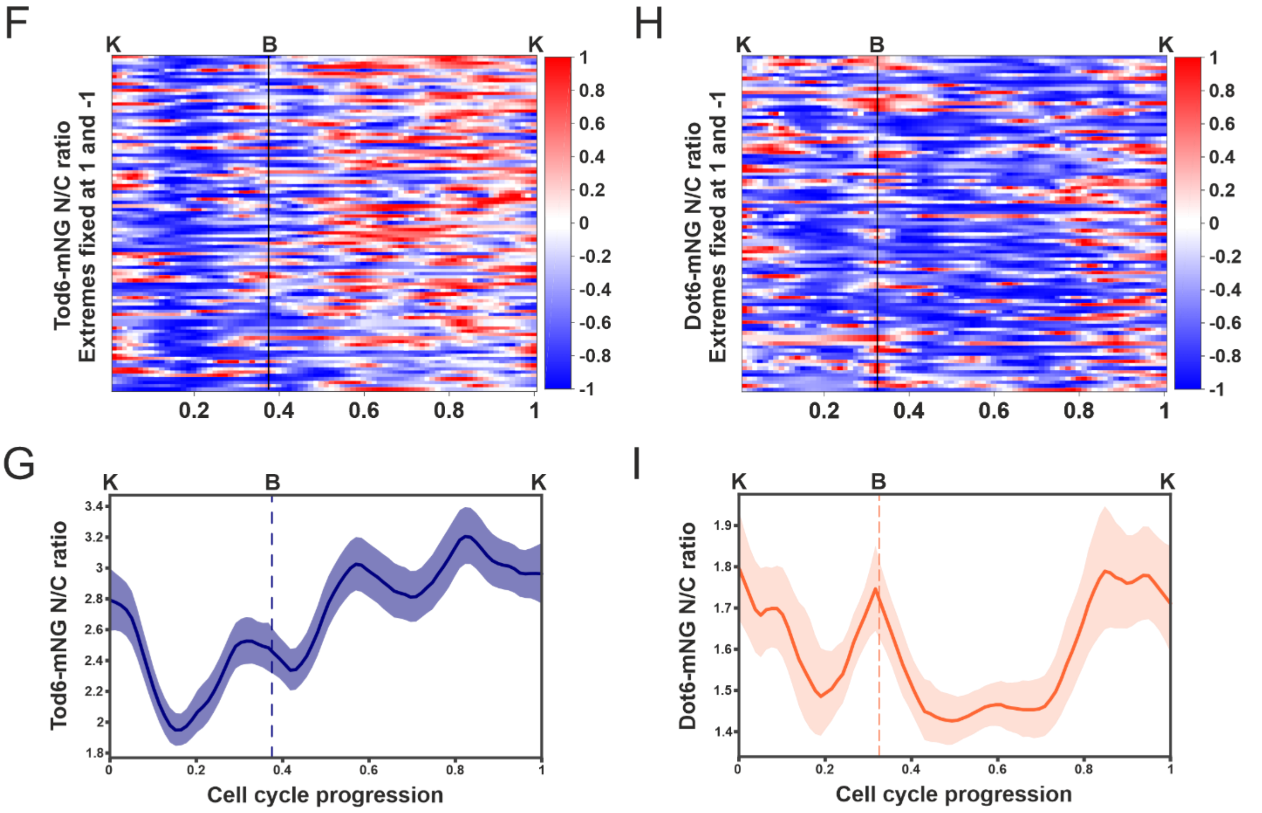

Fig. S2. Related to Figure 2

A. Example images showing a cell that contains Sfp1 tagged with pHtdGFP and Hta2 tagged with mRFP, going through a cell cycle. Upper row shows the signal from Sfp1-pHtdGFP in the GFP channel while the bottom row show the signal from Hta2-mRFP in the RFP channel (for details regarding microscopy cf. Methods). Karyokinesis events, identified by the splitting of the nucleus are annotated. **B.** Example images showing a cell that contains Tod6 tagged with pHtdGFP and Hta2 tagged with mRFP, going through a cell cycle. Upper row shows the signal from Tod6-pHtdGFP in the GFP channel while the bottom row show the signal from Hta2-mRFP in the RFP channel (for details regarding microscopy cf. Methods). Karyokinesis events, identified by the splitting of the nucleus are annotated. **C.** Heatmap of Sfp1-mNeonGreen N/C ratio in individual cell cycles (n = 119). Cell cycle traces were interpolated and aligned as described in Fig.2A. For each cell cycle, the Sfp1 N/C ratio was normalized by assigning its maximum to 1 and its minimum to -1, to facilitate the identification of peaks and troughs. **D.** Average Sfp1 N/C ratio dynamics for the cells shown in **C**. The averages were calculated without normalization of the single-cell data. The bands denote the 95% confidence interval for the mean. **E**. Average cross-correlation (Dunlop *et al.*, 2008) of Whi5 and Sfp1 localization (mean and 95% confidence intervals, n=100 cells). Time delay refers to the shift of the Sfp1 signal against the Whi5 signal. Whi5-mCherry and Sfp1-pHtdGFP were monitored simultaneously in mother cells, focusing in the period a few minutes before cytokinesis and up to the next budding. Whi5 enters the nucleus a few minutes before cytokinesis, and stays there during G1, until mother cells have passed Start (Costanzo *et al.*, 2004). Whi5 and Sfp1 localization was assessed by calculating the standard deviation of the mCherry and GFP signals over the cell pixels, which correlates well with the N/C ratio (Litsios *et al.*, 2019). To calculate the cross-correlation between the Whi5 and Sfp1 localization time series

Journal of Cell Science • Supplementary information

of a mother cell, each time series was first normalized by subtracting its mean and dividing by its standard deviation. Cross-correlations were calculated using the `xcorr` Matlab function. **F**. Heatmap of Tod6-mNeonGreen N/C ratio in individual cell cycles (n = 99). The cell cycles traces were interpolated and aligned as described in Fig.2A. For each cell cycle, the Tod6 N/C ratio was normalized by assigning its maximum to 1 and its minimum to -1, to facilitate the identification of peaks and troughs. **G**. Average Tod6 N/C ratio dynamics for the cells shown in **F**. The averages were calculated without normalization of the single-cell data. The bands denote the 95% confidence interval for the mean. **H.** Heatmap of Dot6-mNeonGreen N/C ratio in individual cell cycles (n = 95) The cell cycles traces were interpolated and aligned as described in Fig.2A. For each cell cycle, the Dot6 N/C ratio was normalized by assigning its maximum to 1 and its minimum to -1, to facilitate the identification of peaks and troughs. **I**. Average Dot6 N/C ratio dynamics for the cells shown in **H**. The averages were calculated without normalization of the single-cell data. The bands denote the 95% confidence interval for the mean

Journal of Cell Science • Supplementary information

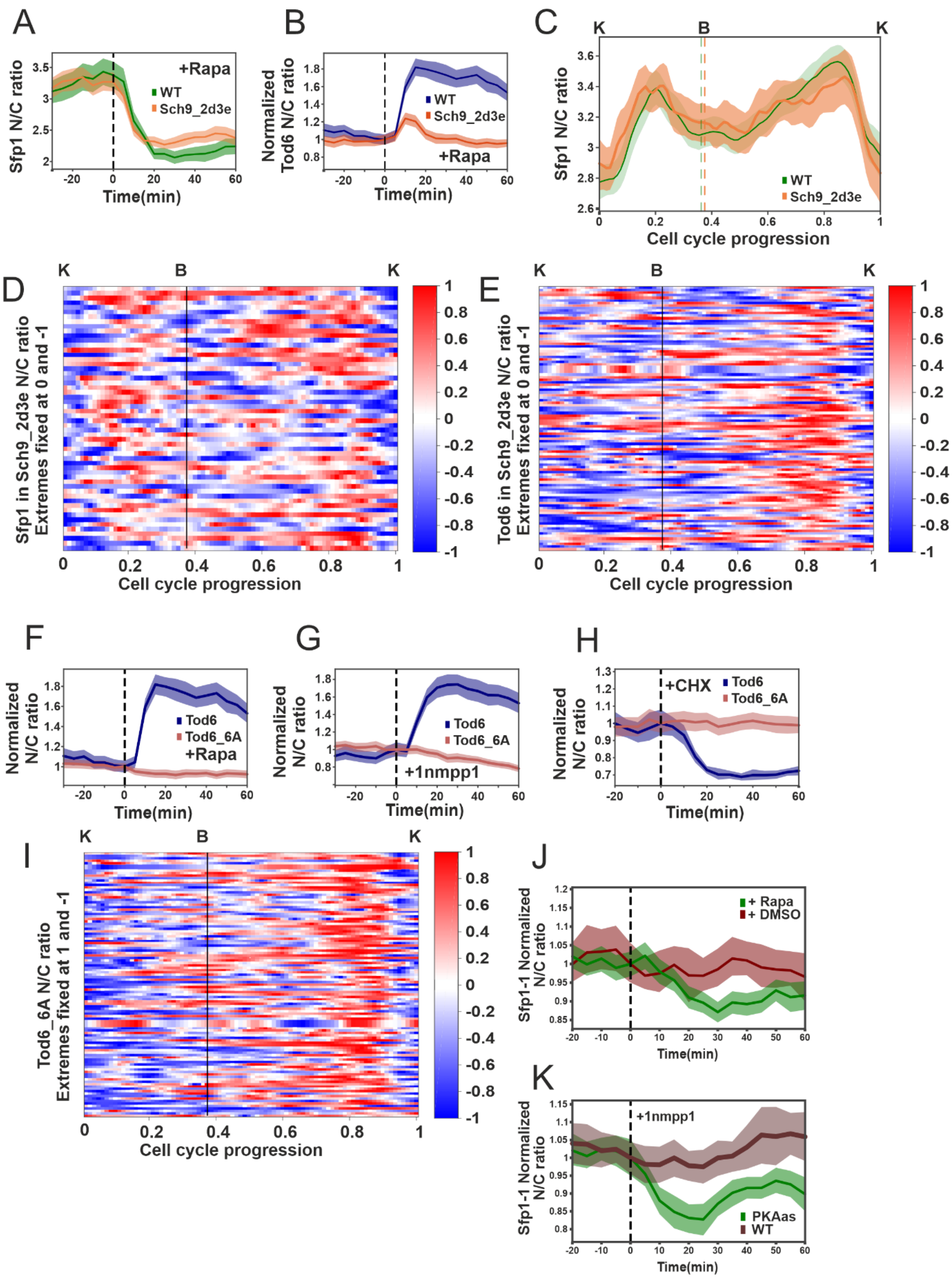

Journal of Cell Science • Supplementary information

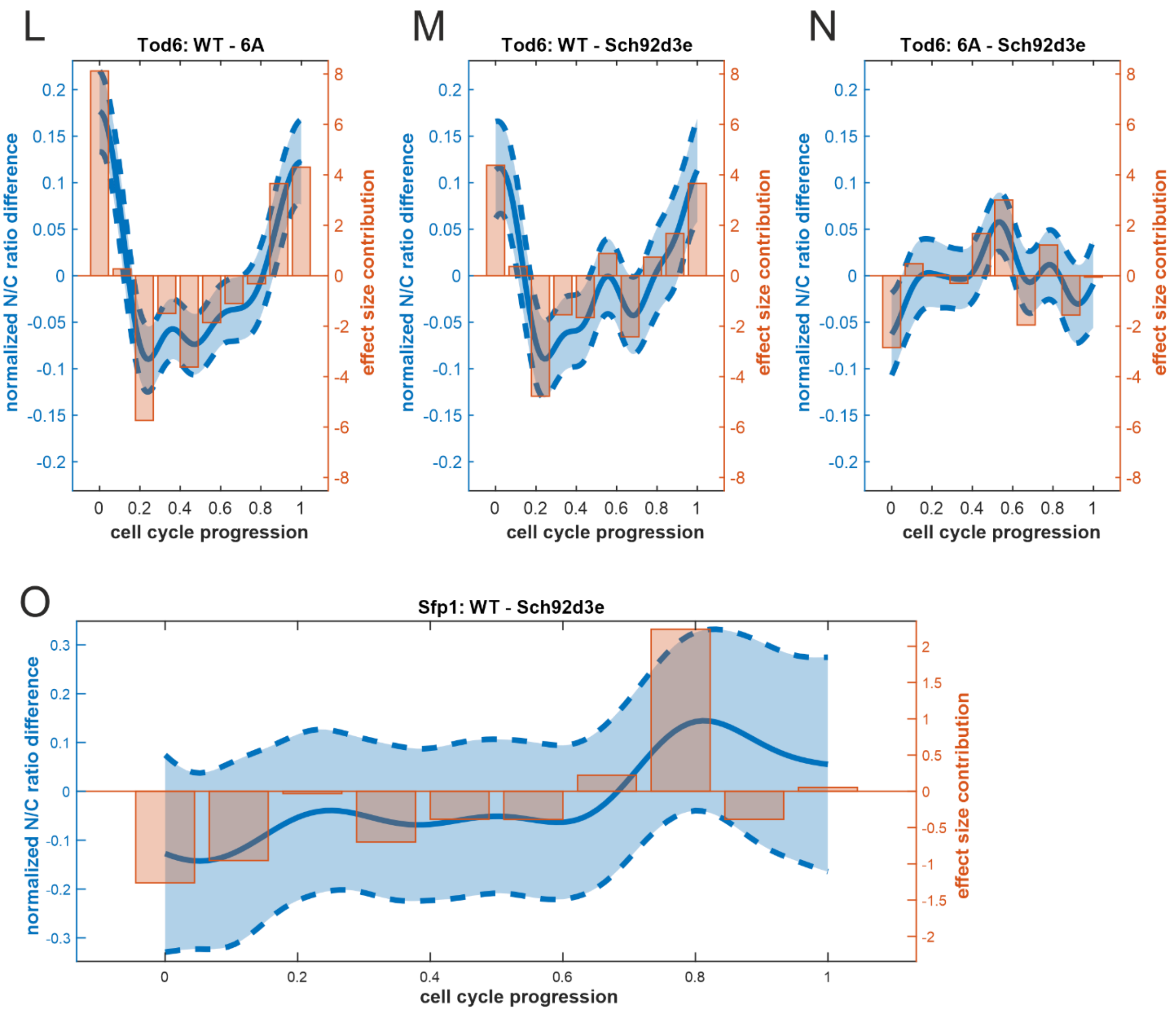

Fig. S3. Related to Figure 3

A. N/C ratio dynamics of Sfp1 in WT(n=56) and in Sch9_2d3e (n=68) cells in response to rapamycin. Rapamycin (200ng/ml final) was added at time t=0. The bands denote the 95% confidence interval for the mean. **B.** Normalized N/C ratio dynamics of Tod6 in WT (n=61) and in Sch9_2d3e (n=76) cells in response to rapamycin. Rapamycin (200ng/ml final) was added at time t=0. Average N/C ratio traces were normalized to their average at t=0 to facilitate comparison. The bands denote the 95% confidence interval for the mean. **C.** Average Sfp1 N/C ratio dynamics in WT (n=149) and in Sch9_2d3e cells (n = 56 cells). The averages were calculated without normalization of the single-cell data. Individual cell cycle traces were interpolated and aligned as described in Fig.2A. Bands denote the 95% confidence interval for the mean. **D**. Heatmap of Sfp1 in Sch9_2d3e cells N/C ratio in individual cell cycles (n = 56). The cell cycle traces were interpolated and aligned as described in Fig.2A. For each cell cycle, the Sfp1 N/C ratio was normalized by assigning its maximum to 1 and its minimum to -1, to facilitate the identification of peaks and troughs. **E.** Heatmap of Tod6 in Sch9_2d3e N/C ratio in individual cell cycles (n = 103). The cell cycle traces were interpolated and aligned as described in Fig.2A. For each cell cycle, the Tod6 N/C ratio was normalized by assigning its maximum to 1 and its minimum to -1, to facilitate the identification of peaks and troughs. **F.** N/C ratio changes of Tod6(n= 61) and Tod6_6A(n=120) in response to rapamycin. Rapamycin (200ng/ml final) was added at time t=0.

Journal of Cell Science • Supplementary information

Average N/C ratio traces were normalized to their average at t=0 to facilitate comparison. The bands denote the 95% confidence interval for the mean. **G.** N/C ratio changes of Tod6(n=63) and Tod6_6A(n=75) in response to addition of 1-NM-PP1 in PKAas cells. 1-NM-PP1 (500nM final) was added at time t=0. Average N/C ratio traces were normalized to their average at t=0 to facilitate comparison. The bands denote the 95% confidence interval for the mean. **H** N/C ratio changes of Tod6(n=56) and Tod6_6A(n=65) in response to addition of CHX. CHX (25ug/ml final) was added at time t=0. Average N/C ratio traces were normalized to their average at t=0 to facilitate comparison. The bands denote the 95% confidence interval for the mean. **I.** Heatmap of Tod6_6A N/C ratio in individual cell cycles (n = 109). Single-cell traces of Tod6_6A localization were aligned and interpolated as described in Fig. 2A. For each cell cycle, the Tod6_6A N/C ratio was normalized by assigning its maximum to 1 and its minimum to -1, to facilitate the identification of peaks and troughs. **J.** N/C ratio changes of Sfp1-1 in response to rapamycin (n=71) and its vehicle DMSO (n=19). Rapamycin (200ng/ml final) and DMSO (0.66% v/v) were at time t=0. Average N/C ratio traces were normalized to their average at t=0. The bands denote the 95% confidence interval for the mean. **K.** N/C ratio changes of Sfp1-1 in response to addition of 1-NM-PP1 in PKAas cells (n=24) and WT cells (n=22). 1-NM-PP1 (500nM final) was added at time t=0. Average N/C ratio traces were normalized at their value to t=0. The bands denote the 95% confidence interval for the mean. **L-N**. Pairwise comparisons of posterior population means for Tod6 localization in WT, Tod6_6A, and Tod6 in Sch9_2d3e cells**.** Single-cell trajectories were normalized by their means, as described in the caption of Fig.3. Means (solid) ± 2 standard deviations (dashed) for each mean difference posterior distribution (left axis, blue curves) and effect size contributions (right axis, orange bars). Effect size contributions provide a visual indication of which points of a sparse time grid produce the strongest deviations between the two population averages. Tail probabilities and effect sizes for each comparison are listed in Table S2. As shown there, the lowest tail probabilities (indicating the greatest differences) are observed when comparing the wild type with the Tod_6A mutant and Tod6 in Sch92d3e. On the other hand, the two mutant strains seem to be more similar to each other (the tail probability is considerably larger). **O.** Comparison of posterior population means for Sfp1 localization in WT and Sch9_2d3e backgrounds**.** Means (solid) ± 2 standard deviations (dashed) for each mean difference posterior distribution (left axis, blue curves) and effect size contributions (right axis, orange bars). The tail probability is ε = 0.581 with an effect size of 8.5, indicating that Sfp1 dynamics does not differ between wild-type and Sch9_2d3e cells.

Journal of Cell Science • Supplementary information

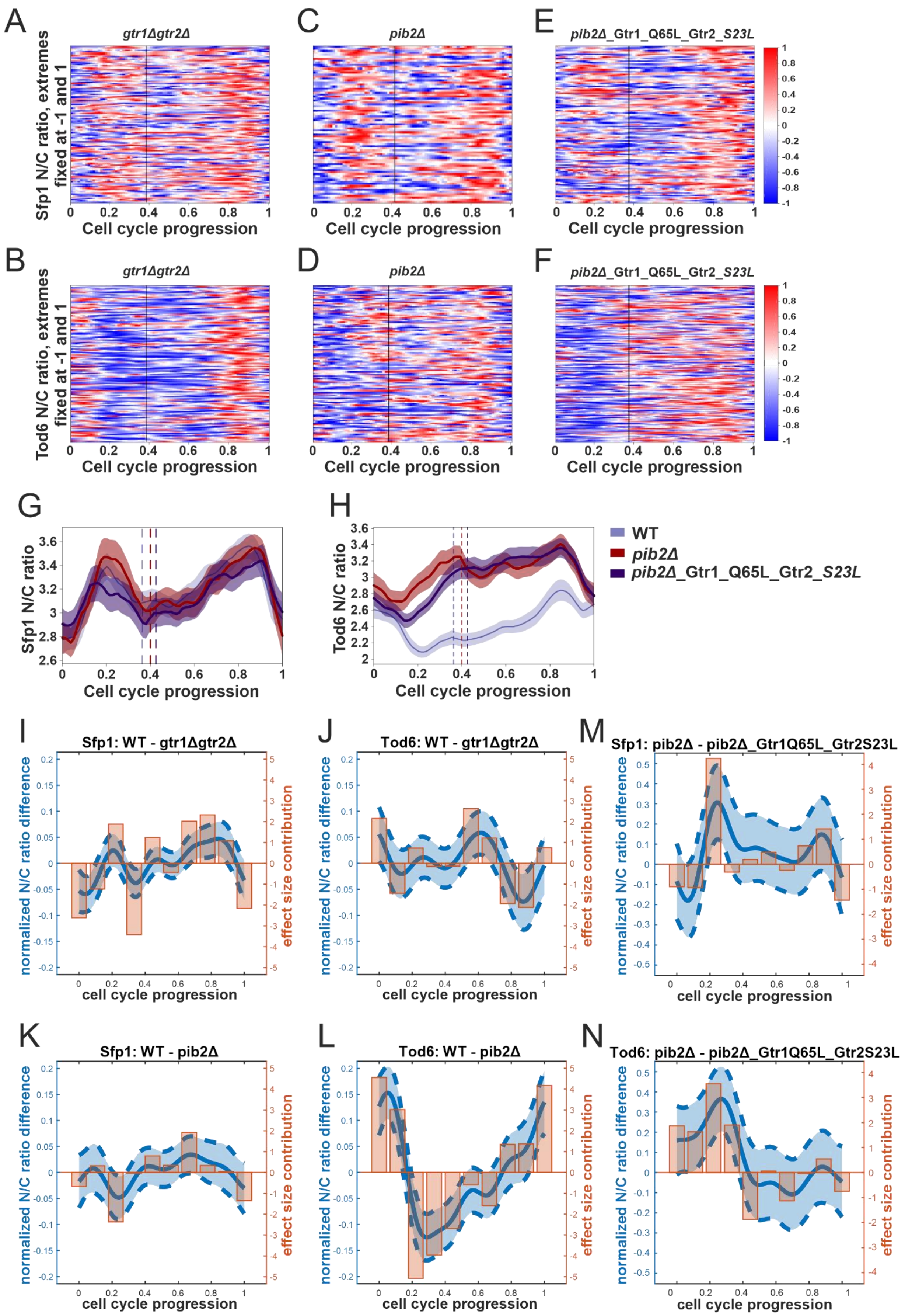

Journal of Cell Science • Supplementary information

Fig. S4. Related to Figure 4

A. Heatmap of Sfp1 N/C ratio in individual cell cycles (n = 122) in *gtr1Δgtr2Δ* cells. Single-cell traces of Sfp1 localization were aligned and interpolated as described in Fig. 2A. For each cell cycle, the Sfp1 N/C ratio was normalized by assigning its maximum to 1 and its minimum to -1. **B.** Heatmap of Tod6 N/C ratio in individual cell cycles (n = 136) in *gtr1Δgtr2Δ* cells. Single-cell traces of Tod6 localization were aligned and interpolated as described in Fig. 2A. For each cell cycle, the Sfp1 N/C ratio was normalized by assigning its maximum to 1 and its minimum to -1. **C.** Heatmap of Sfp1 N/C ratio in individual cell cycles (n = 79) in *pib2Δ* cells. Single-cell traces of Sfp1 localization were aligned and interpolated as described in Fig. 2A. For each cell cycle, the Sfp1 N/C ratio was normalized by assigning its maximum to 1 and its minimum to -1. **D.** Heatmap of Tod6 N/C ratio in individual cell cycles (n = 113) in *pib2Δ* cells. Single-cell traces of Tod6 localization were aligned and interpolated as described in Fig. 2A. For each cell cycle, the Sfp1 N/C ratio was normalized by assigning its maximum to 1 and its minimum to −1. **E.** Heatmap of Sfp1 N/C ratio in individual cell cycles (n = 118) in *pib2Δ*_Gtr1_Q65L_Gtr2_S23L cells. Single-cell traces of Sfp1 localization were aligned and interpolated as described in Fig. 2A. For each cell cycle, the Sfp1 N/C ratio was normalized by assigning its maximum to 1 and its minimum to −1. **F.** Heatmap of Tod6 N/C ratio in individual cell cycles (n = 181) in *pib2Δ*_Gtr1_Q65L_Gtr2_S23L cells. Single-cell traces of Tod6 localization were aligned and interpolated as described in Fig. 2A. For each cell cycle, the Sfp1 N/C ratio was normalized by assigning its maximum to 1 and its minimum to -1. **G.** Average Sfp1 N/C ratio dynamics in WT (n=149), *pib2Δ* (n=79) and *pib2Δ*_Gtr1_Q65L_Gtr2_S23L (n=118) cells. The averages were calculated without normalization of the single-cell data. The cell cycles were interpolated and aligned as described in Fig.2A. The bands denote the 95% confidence interval for the mean. **H.** Average Tod6 N/C ratio dynamics in WT (n=161), *pib2Δ* (n=136) and *pib2Δ*_Gtr1_Q65L_Gtr2_S23L (n=181) cells. The averages were calculated without normalization of the single-cell data. The cell cycles were interpolated and aligned as described in Fig.2A. The bands denote the 95% confidence interval for the mean. **I-N.** Pairwise comparisons of posterior population means for Sfp1 and Tod6 localization in WT, gtr1Δgtr2Δ and pib2Δ backgrounds. Single-cell trajectories were normalized by their means to facilitate comparison of the trends. Means (solid) ± 2 standard deviations (dashed) for each mean difference posterior distribution (left axis, blue curves) and effect size contributions (right axis, orange bars). Effect size contributions provide a visual indication of which points of a sparse time grid produce the strongest deviations between the two population averages. As can be seen from the tail probabilities and effect sizes listed in Table S2, Tod6 trajectories differ most from the wild type in the pib2Δ mutant and considerably less in the gtr1Δgtr2Δ mutant. Sfp1 differences between mutants are not major (tail probabilities > 10^{-6}). **M-N.** Comparison of posterior population means for Sfp1 and Tod6 localization in pib2Δ and pib2Δ_Gtr1Q65L_Gtr2S23L backgrounds**.** Means (solid) ± 2 standard deviations (dashed) for each mean difference posterior distribution (left axis, blue curves) and effect size contributions (right axis, orange bars). The large tail probabilities (>10^{-6}, cf. Table S2) and small effect sizes indicate that Sfp1 and Tod6 dynamics do not differ noticeably between these two strains.

Journal of Cell Science • Supplementary information

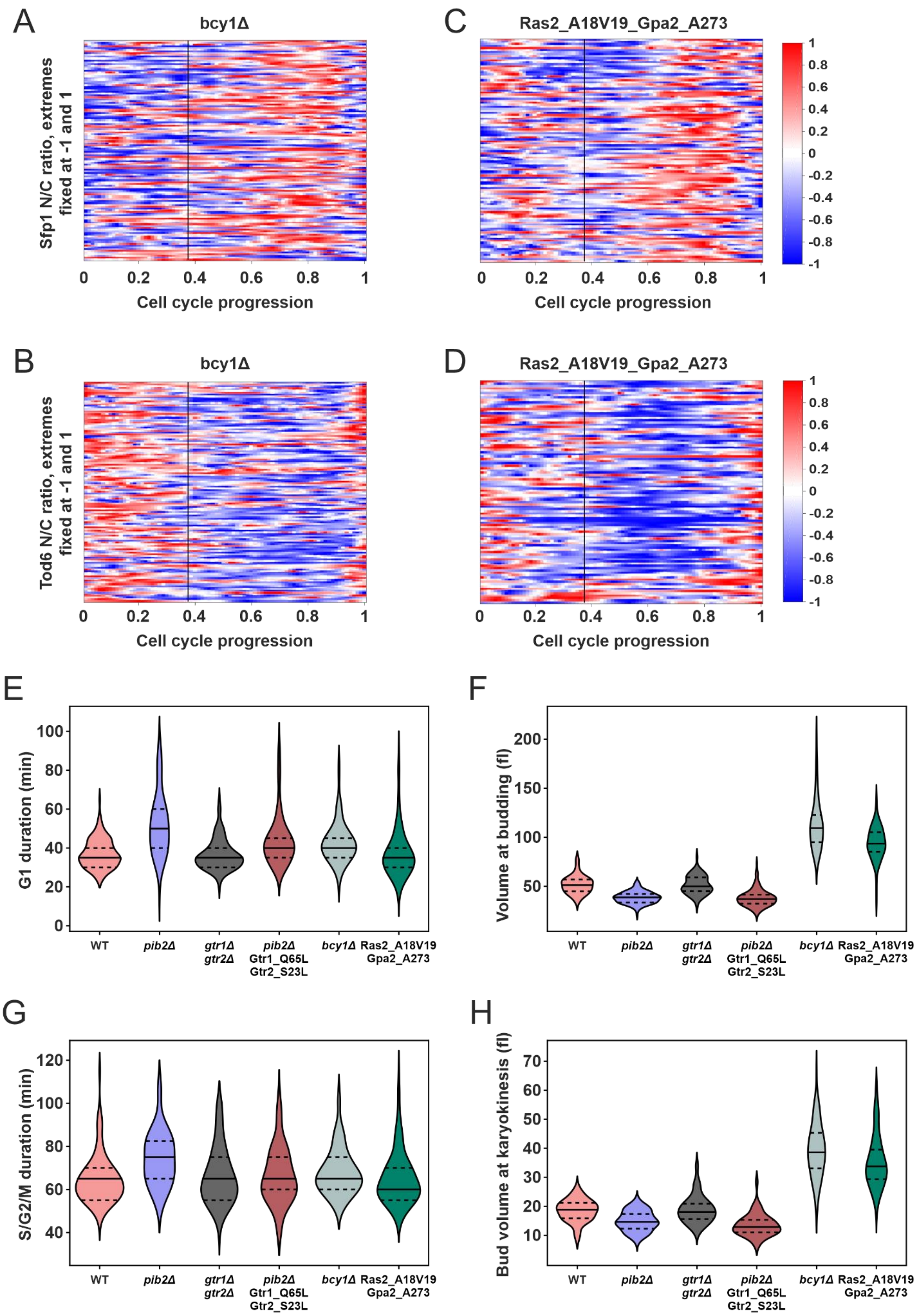

Journal of Cell Science • Supplementary information

I

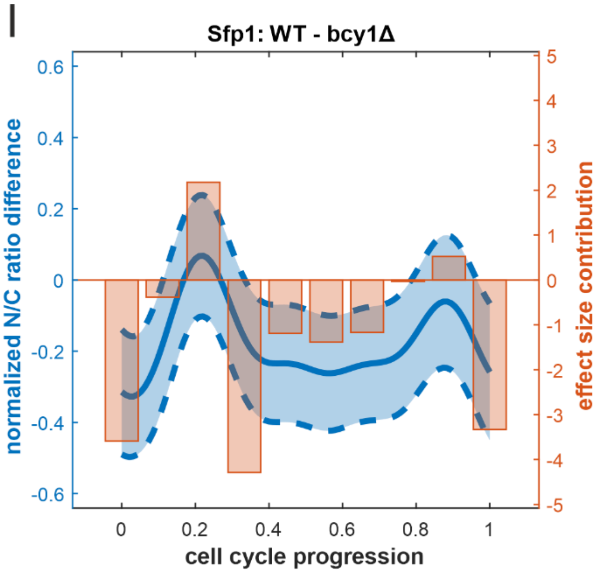

J

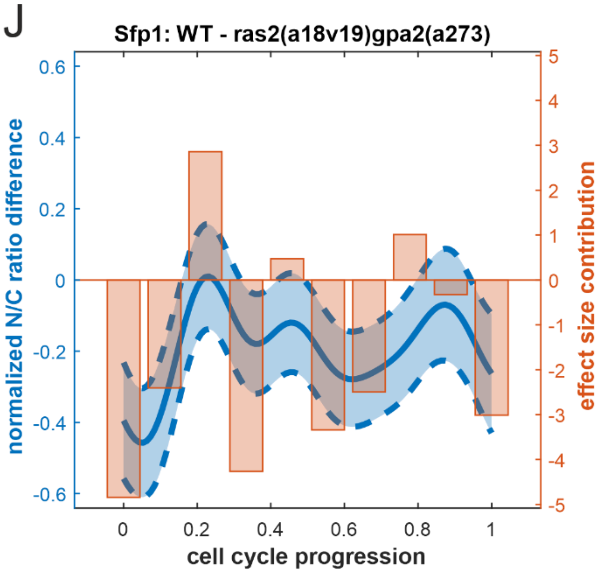

Fig. S5. Related to Figure 5

A. Heatmap of Sfp1 N/C ratio in individual cell cycles (n = 125) in *bcy1Δ* cells. Single-cell traces of Sfp1 localization were aligned and interpolated as described in Fig. 2A. For each cell cycle, the Sfp1 N/C ratio was normalized by assigning its maximum to 1 and its minimum to -1. **B.** Heatmap of Tod6 N/C ratio in individual cell cycles (n = 131) in *bcy1Δ* cells. Single-cell traces of Tod6 localization were aligned and interpolated as described in Fig. 2A. For each cell cycle, the Sfp1 N/C ratio was normalized by assigning its maximum to 1 and its minimum to -1. **C.** Heatmap of Sfp1 N/C ratio in individual cell cycles (n = 111) in Ras2_A18V19_Gpa2_A273 cells. Single-cell traces of Sfp1 localization were aligned and interpolated as described in Fig. 2A. For each cell cycle, the Sfp1 N/C ratio was normalized by assigning its maximum to 1 and its minimum to -1. **D.** Heatmap of Tod6 N/C ratio in individual cell cycles (n = 100) in Ras2_A18V19_Gpa2_A273 cells. Single-cell traces of Tod6 localization were aligned and interpolated as described in Fig. 2A. For each cell cycle, the Sfp1 N/C ratio was normalized by assigning its maximum to 1 and its minimum to -1. **E**. G1 duration distributions of single cells in the wild-type (n = 145), *pib2Δ* (n = 112), *gtr1Δ_ gtr2Δ* (n = 133), *pib2Δ*_Gtr1_Q65L_Gtr2_S23L (n = 183), *bcy1Δ* (n = 135) and Ras2_A18V19_Gpa2_A273 (n = 98) mutants. Median (continuous line) and 25th and 75th percentiles (dashed lines) are also displayed. G1 was defined as the interval between karyokinesis and bud appearance. This definition slightly overestimates the actual G1 duration. Statistical comparison, two-tailed Mann-Whitney test: *pib2Δ* -WT p = $1.5\cdot10^{-17}$,r = 0.61, *gtr1Δ gtr2Δ* -WT p = 0.298, r = 0.15, *pib2Δ* _Gtr1_Q65L_Gtr2_S23L -WT p = $1.1\cdot10^{-7}$, r = 0.34. *bcy1Δ* -WT p = $4.7\cdot10^{-7}$, r = 0.34, Ras2_A18V19_Gpa2_A273 -WT p = 0.25, r = 0.08. **F**. Distributions of volume at the moment of bud appearance in the wild-type (n = 150), *pib2Δ* (n = 122), *gtr1Δ_ gtr2Δ* (n = 136), *pib2Δ* _Gtr1_Q65L_Gtr2_S23L (n = 183), *bcy1Δ* (n = 135) and Ras2_A18V19_Gpa2_A273 (n = 98) mutants. Median (continuous line) and 25th and 75th percentiles (dashed lines) are also displayed. Statistical comparison, two-tailed Mann-Whitney test: *pib2Δ* -WT p = $5.6\cdot10^{-29}$, r = 0.78, *gtr1Δ gtr2Δ* -WT p = 0.89, r = 0.01, *pib2Δ* _Gtr1_Q65L_Gtr2_S23L -WT p = $1.1\cdot10^{-32}$, r = 0.76, *bcy1Δ* -WT p = $5.9\cdot10^{-48}$, r = 0.99,

Ras2_A18V19_Gpa2_A273 -WT $p = 1.06 \cdot 10^{-36}$, $r = 0.95$. **G**. S/G2/M duration distributions in the wild-type (n = 113), *pib2Δ* (n = 103), *gtr1Δ_ gtr2Δ* (n = 113), *pib2Δ* _Gtr1_Q65L_Gtr2_S23L (n = 98), *bcy1Δ* (n = 113) and Ras2_A18V19_Gpa2_A273 (n = 125) mutants. Median (continuous line) and 25th and 75th percentiles (dashed lines) are also displayed. S/G2/M was defined as the interval between bud appearance and karyokinesis. This definition slightly underestimates the actual duration of these phases. Statistical comparison, two-tailed Mann-Whitney test: *pib2Δ* -WT $p = 1 \cdot 10^{-9}$, $r = 0.47$, *gtr1Δ gtr2Δ* -WT $p = 0.46$, $r = 0.15$, *pib2Δ* _Gtr1_Q65L_Gtr2_S23L -WT $p = 0.26$, $r = 0.09$, *bcy1Δ* -WT $p = 0.004$, $r = 0.21$, Ras2_A18V19_Gpa2_A273 -WT $p = 0.93$, $r = 0.29$. **H**. Distributions of bud volume at the moment of karyokinesis in the wild-type (n = 109) and the *pib2Δ* (n = 103), *gtr1Δ_ gtr2Δ* (n = 112), *pib2Δ* _Gtr1_Q65L_Gtr2_S23L (n = 102), *bcy1Δ* (n = 110) and Ras2_A18V19_Gpa2_A273 (n = 123) mutants. Median (continuous line) and 25th and 75th percentiles (dashed lines) are also displayed. Statistical comparison, two-tailed Mann-Whitney test: *pib2Δ* -WT $p = 3.2 \cdot 10^{-11}$, $r = 0.53$, *gtr1Δ gtr2Δ* -WT $p = 0.7$, $r = 0.12$, *pib2Δ* _Gtr1_Q65L_Gtr2_S23L -WT $p = 2.6 \cdot 10^{-17}$, $r = 0.67$, *bcy1Δ* -WT $p = 1.9 \cdot 10^{-35}$, $r = 0.97$, Ras2_A18V19_Gpa2_A273 -WT $p = 1.9 \cdot 10^{-37}$, $r = 0.97$. **I-J.** Pairwise comparisons of posterior population means for Sfp1 localization in WT, bcy1Δ, and Ras2_A18V19_Gpa2_A273 backgrounds. Means (solid) ± 2 standard deviations (dashed) for each mean difference posterior distribution (left axis, blue curves) and effect size contributions (right axis, orange bars). Effect size contributions provide a visual indication of which points of a sparse time grid produce the strongest deviations between the two population averages. For both strains the tail probabilities are quite small ($<10^{-6}$, cf. Table S2) suggesting that the Sfp1 dynamics differs in these strains compared to wild type.

Journal of Cell Science • Supplementary information

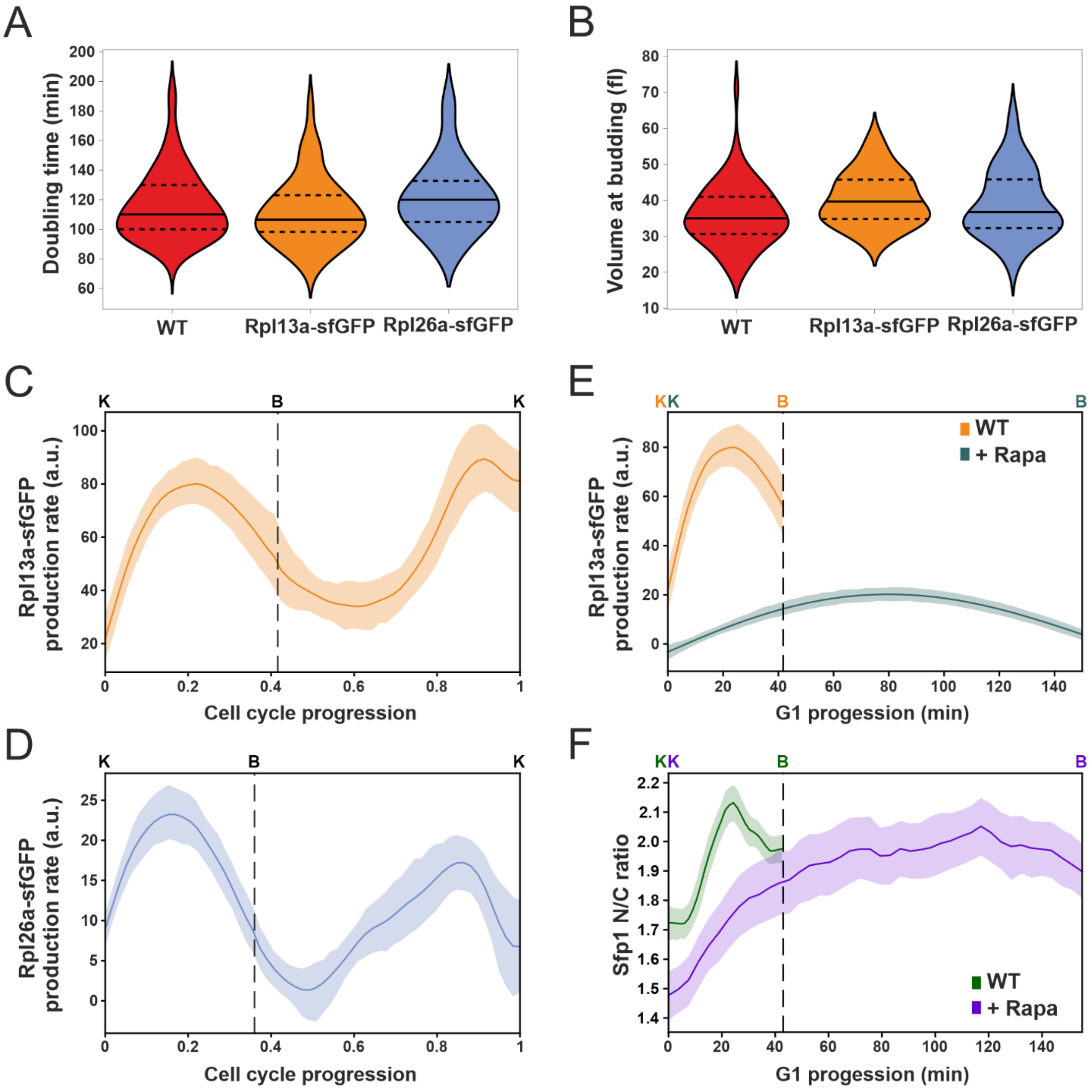

Fig. S6. Related to Figure 6

A. Doubling time (karyokinesis-to-karyokinesis) distributions for single mother cells of the wild type (n = 100), Rpl13a-sfGFP (n = 44) and Rpl26a-sfGFP (n = 48) backgrounds. Median (continuous line) and 25th and 75th percentiles (dashed lines) are also displayed. Statistical comparison, Mann-Whitney test: Rpl13a-WT p-value = 0.36, r = 0.09, Rpl26a-WT p-value = 0.13, r = 0.15 **B.** Distributions of single-cell volumes at budding for mother cells of the wild type (n = 68), Rpl13a-sfGFP (n = 58) and Rpl26a-sfGFP (n = 62) backgrounds. Median (continuous line) and 25th and 75th percentiles (dashed lines) are also displayed. Statistical comparison, Mann-Whitney test: Rpl13a-WT p-value = 0.001, r = 0.33, Rpl26a-WT p-value = 0.07, r = 0.18. **C, D.** Average estimated Rpl13a and Rpl26a synthesis rate (n = 44 cell cycles for Rpl13a, n = 49 cell cycles for Rpl26a). Time series of individual cell cycles were interpolated and aligned as described in Fig.2A. **E**. Average estimated Rpl13a synthesis rate during G1 for WT cells (from

panel C) and cells treated with rapamycin (see Methods for details) (n=52). Time series of individual cell cycles were interpolated as described in Fig.2A and aligned from karyokinesis to budding. The dashed vertical line represents the end of G1 for WT cells. The indicated G1 durations represent the average G1 duration in each condition. **F.** Average Sfp1 N/C ratio dynamics in WT cells (n=149) and cells treated with rapamycin (see Methods for details) (n=112). Time series of individual cell cycles were interpolated as described in Fig.2A and aligned from karyokinesis to budding. The dashed vertical line represents the end of G1 for WT cells. The indicated G1 durations represent the average G1 duration in each condition.

Supplementary Figure References

Costanzo,M. *et al.* (2004) CDK activity antagonizes Whi5, an inhibitor of G1/S transcription in yeast. *Cell*, **117**, 899–913.

Dunlop,M.J. *et al.* (2008) Regulatory activity revealed by dynamic correlations in gene expression noise. *Nat. Genet.*, **40**, 1493–1498.

Litsios,A. *et al.* (2019) Differential scaling between G1 protein production and cell size dynamics promotes commitment to the cell division cycle in budding yeast. *Nat. Cell Biol.*, **21**, 1382–1392.

Journal of Cell Science • Supplementary information

Table S1. List of yeast strains used in this study. List of target sequences and repairing fragments used for Crispr-Cas9 cloning.

Yeast strains		
YSBN6	Steve Oliver lab, Cambridge	YSBN6 *wild type*
YSBN6<br>Hta2-mRFP	This study	YSBN6<br>*HTA2-mRFP1-Ble*
YSBN6<br>Sfp1-pHtdGFP	This study	YSBN6<br>*HTA2-mRFP1-Ble*<br>*SFP1-pHtdGFP-NatMX*
YSBN6<br>Tod6-pHtdGFP	This study	YSBN6<br>*HTA2-mRFP1-Ble*<br>*TOD6-pHtdGFP-NatMX*
YSBN6<br>Sfp1-mNeonGreen	This study	YSBN6<br>*HTA2-mRFP1-Ble*<br>*SFP1-mNeonGreen-NatMX*
YSBN6<br>Tod6-mNeonGreen	This study	YSBN6<br>*HTA2-mRFP1-Ble*<br>*TOD6-mNeonGreen-NatMX*
YSBN6<br>Dot6-mNeonGreen	This study	YSBN6<br>*HTA2-mRFP1-Ble*<br>*DOT6-mNeonGreen-NatMX*
YSBN6<br>Stb3-pHtdGFP	This study	YSBN6<br>*HTA2-mRFP1-Ble*<br>*STB3-pHtdGFP-NatMX*
YSBN6<br>Sfp1-pHtdGFP<br>Whi5-mCherry	This study	YSBN6<br>*SFP1-pHtdGFP-NatMX*<br>*WHI5-mCherry-KanMX*
YSBN6<br>Sfp1-pHtdGFP<br>Tpk1-3as	This study	YSBN6<br>*HTA2-mRFP1-Ble*<br>*SFP1-pHtdGFP-NatMX*<br>*TPK1:tpk1_M164G*<br>*TPK2:tpk2_M147G*<br>*TPK3:tpk3_M165G*
YSBN6<br>Tod6-pHtdGFP<br>Tpk1-3as	This study	YSBN6<br>*HTA2::mRFP1-Ble*<br>*TOD6::pHtdGFP-NatMX*<br>*TPK1:tpk1_M164G*<br>*TPK2:tpk2_M147G*<br>*TPK3:tpk3_M165G*

Journal of Cell Science • Supplementary information

YSBN6<br>Sfp1-pHtdGFP<br>Sch9_2D3E	This study	YSBN6<br>*HTA2-mRFP1-Ble*<br>*SFP1-pHtdGFP-NatMX*<br>*SCH9:sch9^{2D3E}-KanMX*
YSBN6<br>Tod6-pHtdGFP<br>Sch9_2D3E	This study	YSBN6<br>*HTA2-mRFP1-Ble*<br>*TOD6-pHtdGFP-NatMX*<br>*SCH9:sch9^{2D3E}-KanMX*
YSBN6<br>Tod6_6A-pHtdGFP	This study	YSBN6<br>*HTA2-mRFP1-Ble*<br>*HO:TOD6p-tod6^{6A}-pHtdGFP-KanMX*
YSBN6<br>Tod6_6A-pHtdGFP<br>Tpk1-3as	This study	YSBN6<br>*HTA2-mRFP1-Ble*<br>*HO:TOD6p-tod6^{6A}-pHtdGFP-KanMX*<br>*TPK1:tpk1_M164G*<br>*TPK2:tpk2_M147G*<br>*TPK3:tpk3_M165G*
YSBN6<br>Sfp1-1-pHtdGFP	This study	YSBN6<br>*HTA2-mRFP1-Ble*<br>*HO:SFP1p-sfp1-1-pHtdGFP-KanMX*
YSBN6<br>Sfp1-1-pHtdGFP<br>Tpk1-3as	This study	YSBN6<br>*HTA2-mRFP1-Ble*<br>*HO:SFP1p-sfp1-1-pHtdGFP-KanMX*<br>*TPK1:tpk1_M164G*<br>*TPK2:tpk2_M147G*<br>*TPK3:tpk3_M165G*
YSBN6<br>Sfp1-pHtdGFP<br>*gtr1Δ*<br>*gtr2Δ*	This study	YSBN6<br>*HTA2-mRFP1-Ble*<br>*SFP1-pHtdGFP-NatMX*<br>*gtr1Δ*<br>*gtr2Δ*
YSBN6<br>Tod6-pHtdGFP<br>*gtr1Δ*<br>*gtr2Δ*	This study	YSBN6<br>*HTA2-mRFP1-Ble*<br>*TOD6-pHtdGFP-NatMX*<br>*gtr1Δ*<br>*gtr2Δ*
YSBN6<br>Sfp1-pHtdGFP<br>*pib2Δ*	This study	YSBN6<br>*HTA2-mRFP1-Ble*<br>*SFP1-pHtdGFP-NatMX*<br>*pib2Δ::KanMX*
YSBN6<br>Tod6-pHtdGFP<br>*pib2Δ*	This study	YSBN6<br>*HTA2-mRFP1-Ble*<br>*TOD6-pHtdGFP-NatMX*<br>*pib2Δ::KanMX*

Journal of Cell Science • Supplementary information

YSBN6<br>Sfp1-pHtdGFP<br>*pib2Δ*<br>Gtr1_Q65L<br>Gtr2_S23L	This study	YSBN6<br>*HTA2-mRFP1-Ble*<br>*SFP1-pHtdGFP-NatMX*<br>*pib2Δ::KanMX*<br>*GTR1:gtr1_Q65L*<br>*GTR2:gtr2_S23L*
YSBN6<br>Tod6-pHtdGFP<br>*pib2Δ*<br>Gtr1_Q65L<br>Gtr2_S23L	This study	YSBN6<br>*HTA2-mRFP1-Ble*<br>*TOD6-pHtdGFP-NatMX*<br>*pib2Δ::KanMX*<br>*GTR1:gtr1_Q65L*<br>*GTR2:gtr2_S23L*
YSBN6<br>Sfp1-pHtdGFP<br>*bcy1Δ*	This study	YSBN6<br>*HTA2-mRFP1-Ble*<br>*SFP1-pHtdGFP-NatMX*<br>*bcy1Δ::KanMX*
YSBN6<br>Tod6-pHtdGFP<br>*bcy1Δ*	This study	YSBN6<br>*HTA2-mRFP1-Ble*<br>*TOD6-pHtdGFP-NatMX*<br>*bcy1Δ::KanMX*
YSBN6<br>Sfp1-pHtdGFP<br>Ras2_A18V19<br>Gpa2_A273	This study	YSBN6<br>*HTA2-mRFP1-Ble*<br>*SFP1-pHtdGFP-NatMX*<br>*RAS2:ras2_A18V19*<br>*GPA2:gpa2_A273*
YSBN6<br>Tod6-pHtdGFP<br>Ras2_A18V19<br>Gpa2_A273	This study	YSBN6<br>*HTA2-mRFP1-Ble*<br>*TOD6-pHtdGFP-NatMX*<br>*RAS2:ras2_A18V19*<br>*GPA2:gpa2_A273*
YSBN6<br>Rpl13a-sfGFP	This study	YSBN6<br>*HTA2.mRFP1-Ble*<br>*RPL13A.sfGFP-KanMX*
YSBN6<br>Rpl26a-sfGFP	This study	YSBN6<br>*HTA2-mRFP1-Ble*<br>*RPL26A-sfGFP-KanMX*
CRISPR-Cas9 target sequences and repairing fragments		
Cas9 target sequence for Tpk1:<br>TGAAAAATTGCTGAGCATCT	This study	
Cas9 target sequence for Tpk2:<br>GTGATGGATTATATCGAAGG	This study	

Journal of Cell Science • Supplementary information

Cas9 target sequence for Tpk3: GTAATGGCCTACATTGAAGG	This study	
Cas9 target sequence for Gtr1: GAATATGACTCTAAATCTGT	This study	
Cas9 target sequence for Gtr2: TGGTTTTGTTGATGGGCGTA	This study	
Cas9 target sequence for Ras2: TACAAGCTAGTCGTCGTTGG	This study	
Cas9 target sequence for Gpa2: CTTAATATGACCTGCTGGGT	This study	
gtr1Δ repairing fragment: AGGTATCTTACACAGGAGTGAAGGC CATCAAAATCACGTTTATCAATCGAC AATTTAGTACTGAGGTGAGTAGACG AAACATTCGGCAATTGAGTGTTTGC GGGGCATAAGAATTATAAA	This study	
gtr2Δ repairing fragment: ACCGATTAACATCCACAGATTAACAA AACTCCAGGACAACGGTACTAATAC ACATACAACAAGACGTAAGGCATGA AAATATTAGGGTATATAGATACATAT TGAAAATGATAGTAGAGC	This study	
Gtr1_Q65L repairing fragment: GCCACCATTGATGTAGAGCACTCCC ATTTGAGATTTCTTGGGAATATGACT CTTAACCTCTGGGACTGTGGTGGGC TGGACGTGTTTATGGAGAATTATTTC ACCAAGCAAAAAGACCAC	This study	
Gtr2_S23L repairing fragment: CCAGGACAACGGTACTAATACACAT ACAACATGAGTTTAGAGGCTACAGA TTCCAAGGCAATGGTTTTGTTGATG GGCGTAAGAAGATGTGGAAAATTAT CCATTTGTAAAGTTGTTTTT	This study	
Ras2_A18V19 repairing fragment: GAATTGAAAGGAGATATACAGAAAA AAAAATGCCTTTGAACAAGTCGAAC ATAAGAGAGTACAAGCTAGTTGTTG TCGGAGCTGTTGGTGTTGGTAAATC TGCTTTGACCATACAATTGACCCAAT CGCACTTTGTAGATGAATACGATCC CACAATTGA	This study	

Journal of Cell Science • Supplementary information

Gpa2_A273 repairing fragment: CGAAGTTCTATCTAATGGACTCGAC TCCTTACTTCATGGAAAATTTCACCA GGATCACTTCGCCCAATTACAGACC CACTCAACAAGACATATTAAGATCG GCTCAGATGACGTCAGGGATTTTTG ACACCGTCATTGATATGGGGTCGGA TATCAAGATGCATATTTACGACGTG GGTG	This study	
Tpk1_M164G repairing fragment: TTTCTATCGTAACACATCCGTTTATT ATTAGAATGTGGGGGACTTTCCAAG ATGCTCAGCAAATTTTCATGATTGGT GATTATATTGAAGGTGGAGAATTGTT TTCTTTGTTAAGGAAATCCCAAAGAT TTCCCAATCCTGTCGCTAAATTTTAC GCAGCGGAAGTTTGTTTAGCTTTGG AGTACTTGCATAGCAAGGACATTATT TATAGGGATTTGAA	This study	
Tpk2_M147G repairing fragment: TGATTAGAATGTGGGGTACGTTTCA AGATGCTAGGAATATCTTTATGGTG GGTGATTATATAGAGGGTGGTGAAC TTTTCTCGTTACTGAGAAAGTCACAA AGATTTCCTAATCCTGTAG	This study	
Tpk3_M165G repairing fragment: CATCATTCGAATGTGGGGAACGTTC CAAGATTCTCAGCAAGTTTTCATGGT AGGTGACTACATCGAG GGTGGTGAATTATTTTCTTTACTACG TAAATCTCAAAGATTTCCCAACCCAG T	This study	

Journal of Cell Science • Supplementary information

Table S2. Comparison results for single-cell time series of Sfp1 and Tod6 localization generated from the different mutant strains described in the main text, and described in the Supplementary Figures. A detailed mathematical description of our comparison approach as well as the computed metrics, can be found in the Methods section of the main text. Briefly, the tail probability indicates how significant the difference between the two population averages is (values larger than 10^{-6} indicate small or no difference), while the effect size quantifies the magnitude of the difference. Tail probabilities smaller than 10^{-6} (indicating large differences) and their corresponding effect sizes are highlighed in red. Larger tail probabilities and corresponding effect sizes are highlighed in blue.

Compared strains	Tail probability (ε)	Effect size	Suppl. Figure
Tod6: WT - 6A	2.40E-27	150.9	S3
Tod6: WT - Sch92d3e	3.32E-11	70.65	S3
Tod6: 6A - Sch92d3e	0.00179	28.0	S3
Tod6: WT - gtr1Δgtr2Δ	0.00647	24.5	S4
Tod6: WT - pib2Δ	1.66E-17	102.6	S4
Tod6: pib2Δ - pib2Δ_Gtr_locked	0.00177	28.1	S4
Sfp1: WT - gtr1Δgtr2Δ	1.28E-5	40.7	S4
Sfp1: WT - pib2Δ	0.251	12.5	S4
Sfp1: pib2Δ - pib2Δ_Gtr_locked	0.00610	24.6	S4
WT - bcy1Δ	1.05E-7	52.2	S5
WT - Ras2_A18V19_Gpa2_A273	1.08E-13	83.4	S5

Journal of Cell Science • Supplementary information
