# Supplementary material for: TORC1 and PKA activity towards ribosome biogenesis oscillates in synchrony with the budding yeast cell cycle
Source: J Cell Sci. 2022 Sep 28;135(18):jcs260378. doi: 10.1242/jcs.260378 (PMC9658999; doi:10.1242/jcs.260378)
Supplement: Supplementary file 1 [file joces-135-260378_review_history.pdf]

TORC1 and PKA activity towards ribosome biogenesis oscillates in synchrony with the budding yeast cell cycle

Paolo Guerra, Luc-Alban P. E. Vuillemenot, Yulan B. van Oppen, Marije Been and Andreas Milias-Argeitis

DOI: 10.1242/jcs.260378

Editor: David Glover

Review timeline

Submission to Review Commons:	3 June 2021
Submission to Journal of Cell Science:	23 June 2022
Accepted:	11 July 2022

Reviewer 1

Evidence, reproducibility and clarity

Summary:

In this paper, Guerra and co-workers analyze the dynamics of the TORC1 and PKA signaling pathways during the cell cycle of the budding yeast Saccharomyces cerevisiae. By using the technique of single-cell time-lapse fluorescence microscopy, the authors monitor the nucleo/cytosolic localization of transcriptional activators/repressors as a readout for the activation of the signaling pathways, showing that both TORC1 and PKA activity peaks in G1 and is low at the onset of budding and in M phase.

Major comments:

The paper is generally convincing. However, additional experiments are necessary to fully support the authors' conclusion. In Figure 1F, the authors show that inhibition by 1NM-PP1 of an ATP analog-sensitive PKA causes a drop of the N/C ratio of Sfp1 and a raise of the N/C ratio of Tod6 which are remarkably similar to those caused by rapamycin. Here, I feel that a proper control, like the treatment of a wt strain with 1NM-PP1, is certainly needed to demonstrate that the effect is not due to off-targets of the drug, for example some poor kinase in terms of ATP affinity (like TORC1 itself).

In Figure 3, the authors show the de-regulation of the cell cycle features of Tod6 localization in the presence of a Sch9 mutant and of a Tod6 allele where all the known Sch9/PKA phosphosites are mutated to alanine. The two mutants show basically the same phenotype. However, shouldn't the two mutants show opposing phenotypes, since the Tod6-6A mutant is non-phosphorylatable while the Sch9-2D3E mutant is constitutively active? The authors should comment on that.

The authors claim that the TORC1/PKA activity peaks are somehow reflected in ribosome biogenesis dynamics. To prove a causative relation and not just a temporal correlation, some experiment is undoubtedly needed. An example could be the treatment with sub-lethal concentrations of rapamycin, which allow the cells to cycle. In these conditions, if a causative relation exists, the peaks of Sfp1 N/C ratio and RP production should be coordinately blunted.

As for the data presentation, it seems evident that the baseline experiments (wt profiles of N/C rations of Sfp1/Tod6) were carried out once and then repeatedly re-plotted in several figures. Even if the profile shown is the average of more than 100 cells, it is not good practice to show the same controls for multiple figures.

© 2022. Published by The Company of Biologists under the terms of the Creative Commons Attribution License (https://creativecommons.org/licenses/by/4.0/).

Moreover, the authors show in the supplementary figures (Figure S1 D-E) that the vehicle of rapamycin (DMSO) causes some transient effect. I think that these data should be in the main figures, alongside the profiles of the drug-treated cells as it's usually the case with all the other techniques (growth kinetics, western blots, etc.).

Minor comments:

There is a mistake in the reference of Di Talia et al., as it is mentioned both in the text and in the references section as Talia et al., while the complete last name is Di Talia.
The nomenclature of yeast genes and mutants throughout the paper is rather unusual. Examples are the deletion mutants reported as ?Abc1, while the general consensus is to write them as abc1? (all lowercase and italic). Point mutations are sometimes reported with superscript text, sometimes with underscores separating them from the gene/protein name. An example is Figure 3, where the same mutant is labeled in two different ways in panels C and D.

Significance

The finding that the activity of TORC1/PKA signaling pathways oscillates with the cell cycle is interesting for the field because it could stimulate the investigation of the molecular cues driving the oscillation, imagining the activatory signals (nutrients) as constitutively present in the medium.

The paper is a seldom example of evaluation of the activity of traditionally metabolism- related signaling pathways in the context of cell cycle. I think there should be more of this kind of studies, since some molecular phenotypes, maybe confined in a certain cell cycle phase, might be sometimes masked in the study of asynchronous populations.

Reviewer 2

Evidence, reproducibility and clarity

In this paper, Guerro et al. investigate the impact of the major growth regulatory pathways PKA and TOR on cell cycle progression. They use fluorescence microscopy and PKA and TOR activation state reporters to determine their dynamic properties during the cycle. First, they confirm that Sfp1-GFP and Tod6-GFP are valid fluorescent reporters of the activation state of PKA and TOR using drugs that target these two pathways, in agreement with previous studies. Then, they show that, while the localization of the two reporters is coincidant in G1 phase, they seem to give contradictory signals in G2/M, suggesting a more complex regulation in this phase of the cycle. To further investigate the regulation of PKA and TOR in G2/M, the authors use several genetic perturbations of PKA and TOR signaling. While confirming the role of several known players in PKA and TOR signaling, these manipulations do not fully elucidate the origin of the dynamic pattern of Tod6 relocalization during the cycle. In the last part of the paper, the authors focus on the functional consequences of genetic perturbations of PKA and TOR by looking at coordination between growth and division. They show that over-activation of PKA (which leads to a faster volume increase rate) induces a larger cell-cell variability in cell size, suggesting that the coupling between growth and division is partially abolished. Finally, the authors use reporters of ribosome synthesis to show that there are two peaks of synthesis during the cycle in G1 and G2/M.

Major comments:
1)The study is technically sound and corresponds to current standards in terms of data transparency and rigor. However, some additional elements could improve the robustness of the conclusions: statistical test, raw images, time traces of single cells to illustrate the phenomena presented, see below.
2)Similarities and differences in the regulation of localization between Sfp1 and Tod6 are intriguing and suggest looking at other transcription factors that are also regulated by PKA and TOR (Dot6, Maf1...). Extending the analysis, at least in a WT context, could clarify why there are divergences at the G2 phase. The observations suggest either that Tod6 and/or Sfp1 are not good indicators of TOR/PKA activity (at least in G2/M), or that more complex regulation is stake (see below). The authors tend to prefer the former interpretation, but there is no clear experimental evidence that supports it.

© 2022. Published by The Company of Biologists under the terms of the Creative Commons Attribution License (https://creativecommons.org/licenses/by/4.0/).

3)It could be useful to distinguish between mother and daughter cells when performing the synchronization of temporal signals. Substantial differences in mother vs daughter G1 durations may blur the average dynamics of the localization of Tod6/Sfp1, and might therefore change the interpretation of the data.
4)This work provides interesting data confirming the peak of synthesis in G1 phase previously established by several groups (including the authors) and suggests that there is another peak of synthesis in G2/M. It would be interesting for the authors to discuss the relevance and significance of this second peak and why it has not been observed in their previous study (Litsios et al, 2019)

Minor comments:

1) please show single-cell traces along with raw images corresponding to experiments in Fig 1C-J

2) please display consistent Y scales to appreciate differences in amplitudes on Fig 1, especially since CHX induces an effect opposite to other drugs.

3) please indicate the strain background on each panel if different between Fig. 1C-E and 1F

4) Fig 3C: please show the statistical test to confirm the significance of the difference between mutants

5) On Fig 4, the Tod6-related panels are on the left column, yet they are on the right column on Fig 5. It would be more clear to have a consistent layout of the panels.

6) "At the same time" on page 16: "the" is missing

7) Fig. 6: statistical tests are missing. Quantification of variability is missing (CV values, or even better, Fano factor would be necessary to compare variability in cell size since distributions have strongly different mean values)

8) Fig. 7: Sequence of images of cells at different time points is needed to assess the fluorescence signal and the coordination with the cell cycle.

9) Fig S2 should come before S1, because it described the methodology described in Figure S1

10) Fig 2A: Please provide images corresponding to fluorescently tagged Sfp1 and Tod6 on this sequence of images

11) Page 9: "allbfeatures of the wild-type Tod6 N/C ratio during G1, budding and karyokinesis had disappeared" : it's a bit difficult to compare "features" by visual inspection of the data, knowing that statistical significance is not provided

Significance

The study is interesting because it provides a new angle (namely single cell dynamics) on the major growth regulation mechanisms, mainly studied using bulk measurements so far. The regulation of metabolism during the cell cycle remains a fundamental and still poorly characterized question. This study provides new insights, although it does not clearly explain how PKA and TOR regulate the cell cycle (and potentially vice versa), and some questions persist regarding the interpretation of the observed dynamics of Tod6 and Sfp1 reporters. That being said, it provides useful confirmation of the role played by genes part of the PKA/TOR pathways on specific transcription factors and its impact on cell cycle progression and the coordination of size and cell division.

© 2022. Published by The Company of Biologists under the terms of the Creative Commons Attribution License (https://creativecommons.org/licenses/by/4.0/).

Reviewer 3

Evidence, reproducibility and clarity

The paper by Guerra et al describes the changes in the nuclear localization of Sfp1 and Tod6 as reporters of TOR and PKA, respectively, during the cell cycle in budding yeast. The experiments are correctly designed and explained, and the results are interesting in the context of the mechanisms that coordinate cell growth and proliferation. However, in my view, the main conclusion of this work should be substantiated by connecting their observations further with a few key experiments as detailed below.

1. Sfp1 nuclear localization seems to be maximal around Start. The progressive increase during G1 fits with the notion proposed by Litsios et al (2019) that an autonomous metabolic cycle has an important role in execution of Start by stimulating Cln3 synthesis. In this regards, the authors state that "our results may therefore explain the cause of the observed pulses in protein synthesis and Cln3 concentration during G1". However, this statement would require a correlative analysis of Sfp1 with Whi5 localization from birth to budding.
Also, the behavior of Sfp1 localization should be tested in genetically G1 arrested cells to rule out Cdk-dependent effects.

2. The authors clearly show that changes in Tod6 localization during G2/M do not depend on TORC1 or PKA activity. Furthermore, Spf1 and Tod6 N/C ratios show complementary patterns in G1, but similar in G2/M, suggesting that nutrient-independent and perhaps Cdk- dependent signals operate on the mechanisms that control their localization during G2/M. This key question could be easily tested in a cdc28-4 mutant, perhaps using cdc20-arrested cells for comparison.

3. Bcy1 mutant cells are much larger, which likely explains the observed differences in volume growth rate when compared to wt. The same reasoning in the opposite sense applies to pib2 cells. Thus, the conclusion that "alterations in the TORC1 and PKA cell cycle activity pattern perturb the coupling between cell cycle and growth" is not supported by these data, which should be removed or complemented by independent approaches.

4. RP synthesis patterns are interesting and coherent with the observed changes in Spf1 and Tod6 localization. Rate values are plotted per volume unit, which is correct but does not reflect the rate change in volume growth. I would suggest the authors add a plot with the overall rate data.

Significance

This work addresses the mechanisms that coordinate cell growth and proliferation in a relevant eukaryotic model and provides interesting observations contributing to the growing evidence that the cell cycle receives many independent inputs from parallel oscillating processes, a still thought-provoking topic in cell biology.

Reviewer keywords: cell cycle, growth, systems biology

Author response to reviewers' comments

1. General Statements

We would like to thank the reviewers for their thoughtful comments and constructive feedback. Below, we respond to the individual comments of each reviewer, and describe the actions taken to address each of their comments. Reviewer comments are displayed in *blue italic* font, and our replies in regular font. Actions taken and items added to the manuscript (or modified) are highlighted in **bold**. Finally, new or modified parts of the main text are annotated with red font.

© 2022. Published by The Company of Biologists under the terms of the Creative Commons Attribution License (https://creativecommons.org/licenses/by/4.0/).

***Note**: Due to formatting constraints, the supplementary figures of the revised manuscript (whose numbers are mentioned in the reviewer response letter) had to be re-ordered and re-grouped in the published version of the manuscript. Therefore, Figs. S1, S2 and S3 (revised version) correspond to Figs. S1 (published version); Figs. S4 and S5 (revised version) correspond to Fig. S2 (published version); Figs. S6, S7, S12 and S15 (revised version) correspond to Fig. S3 (published version); Figs. S8, S13 and S16 (revised version) correspond to Fig. S4 (published version); Figs. S9, S10 and S14 (revised version) correspond to Fig. S5 (published version); and Fig. S11 (revised version) corresponds to Fig. S6 (published version).*

2. Point-by-point description of the revisions

Reviewer 1

The paper is generally convincing. However, additional experiments are necessary to fully support the authors' conclusion. In Figure 1F, the authors show that inhibition by 1NM-PP1 of an ATP analog-sensitive PKA causes a drop of the N/C ratio of Sfp1 and a raise of the N/C ratio of Tod6 which are remarkably similar to those caused by rapamycin. Here, I feel that a proper control, like the treatment of a wt strain with 1NM-PP1, is certainly needed to demonstrate that the effect is not due to off-targets of the drug, for example some poor kinase in terms of ATP affinity (like TORC1 itself).

We have carried out the controls requested by the reviewer, and present them on **Fig. 1**. 1NM- PP1 has some clear off-target activity, but its effect on PKA-as strains can be distinguished from its effect on the wild-type.

In Figure 3, the authors show the de-regulation of the cell cycle features of Tod6 localization in the presence of a Sch9 mutant and of a Tod6 allele where all the known Sch9/PKA phosphosites are mutated to alanine. The two mutants show basically the same phenotype. However, shouldn't the two mutants show opposing phenotypes, since the Tod6-6A mutant is non-phosphorylatable while the Sch9-2D3E mutant is constitutively active? The authors should comment on that.

The common feature of these two mutants is that TORC1-dependent signals cannot propagate to Tod6: in the case of *Tod6-6A*, this happens because all known phosphosites affected by Sch9/PKA have been mutated to alanine. In the case of *Sch9-2D3E*, the phosphomimetic mutations on the protein should prevent changes in TORC1 activity from propagating to Tod6 via Sch9. As Figs. 3B and D show, the N/C profiles of Tod6 over the cell cycle are very similar in both mutants, as expected from the fact that in both mutants Tod6 no longer responds to changes in TORC1 activity. However, we should note that in Figs. 3B and D the traces are normalized to their means in order to facilitate comparison of patterns. As shown on Fig. 3C, the average N/C ratio of Tod6 actually differs between the two mutants, as a consequence of differences in the phosphorylation of the protein (*Tod6-6A* cannot be phosphorylated, whereas wild-type Tod6 in the *Sch9-2D3E* can be phosphorylated). Therefore, the phenotypes of the two mutants are not the same.

The authors claim that the TORC1/PKA activity peaks are somehow reflected in ribosome biogenesis dynamics. To prove a causative relation and not just a temporal correlation, some experiment is undoubtedly needed. An example could be the treatment with sub-lethal concentrations of rapamycin, which allow the cells to cycle. In these conditions, if a causative relation exists, the peaks of Sfp1 N/C ratio and RP production should be coordinately blunted.

Following the reviewer's suggestion, we quantified ribosomal protein (RP) synthesis rates in mother cells treated with a sub-lethal dose of rapamycin, with the caveat that inhibition of TORC1 by rapamycin has many secondary effects that are hard to predict, since TORC1 controls many different aspects of cell growth. As can be seen on **Fig. S11E,F**, these cells have a considerably longer G1 phase compared to the wild-type. Moreover, the RP synthesis rate in rapamycin-treated cells shows a blunted peak during G1 compared to wild-type, and this behavior is in good agreement with the correspondingly blunted peak of Sfp1 localization.

We also observed that RP expression appears to fully stop during S/G2 in rapamycin-treated cells, as shown in the plot below. What is more, RP abundance seems to *decrease* in S/G2, as can be seen by the slight decrease in the total GFP fluorescence of cells in that phase. This dramatic change in RP expression in S/G2 could be due to a combination of altered transcriptional regulation and/or the

© 2022. Published by The Company of Biologists under the terms of the Creative Commons Attribution License (https://creativecommons.org/licenses/by/4.0/).

induction of autophagy, which rapamycin is known to cause. Although we found this observation very interesting, we avoided to report it in the main text because it would add unnecessary complexity while not offering any concrete insights. We hope to follow up on this finding in our future work.

***NOTE**: We have removed unpublished data that had been provided for the referees in confidence.*

As for the data presentation, it seems evident that the baseline experiments (wt profiles of N/C rations of Sfp1/Tod6) were carried out once and then repeatedly re-plotted in several figures. Even if the profile shown is the average of more than 100 cells, it is not good practice to show the same controls for multiple figures.

We understand the concern of the reviewer, and apologize for any confusion caused. The overlay of the wild-type profiles to the mutant ones was only meant to provide a visual aid to the reader, and facilitate the comparison of features between profiles. We have now **clarified this point in the figure captions**, and made the wild-type profiles fainter. We hope that these actions address the reviewer’s concern.

Moreover, the authors show in the supplementary figures (Figure S1 D-E) that the vehicle of rapamycin (DMSO) causes some transient effect. I think that these data should be in the main figures, alongside the profiles of the drug-treated cells as it's usually the case with all the other techniques (growth kinetics, western blots, etc.).

Following, the advice of the reviewer, we have now **incorporated these control experiments into the main text figure**.

There is a mistake in the reference of Di Talia et al., as it is mentioned both in the text and in the references section as Talia et al., while the complete last name is Di Talia. The nomenclature of yeast genes and mutants throughout the paper is rather unusual. Examples are the deletion mutants reported as ΔAbc1, while the general consensus is to write them as abc1Δ (all lowercase and italic). Point mutations are sometimes reported with superscript text, sometimes with underscores separating them from the gene/protein name. An example is Figure 3, where the same mutant is labeled in two different ways in panels C and D.

We apologize for the incorrect nomenclature of genes and mutants. We have **corrected the notation throughout the paper**, and also **fixed inconsistencies in the references**.

Reviewer 2

1) The study is technically sound and corresponds to current standards in terms of data transparency and rigor. However, some additional elements could improve the robustness of the conclusions: statistical test, raw images, time traces of single cells to illustrate the phenomena presented, see below.

As we hope to illustrate below, we have tried to address all suggestions of the reviewer.

2) Similarities and differences in the regulation of localization between Sfp1 and Tod6 are intriguing and suggest looking at other transcription factors that are also regulated by PKA and TOR (Dot6, Maf1...). Extending the analysis, at least in a WT context, could clarify why there are divergences at the G2 phase. The observations suggest either that Tod6 and/or Sfp1 are not good indicators of TOR/PKA activity (at least in G2/M), or that more complex regulation is stake (see below). The authors tend to prefer the former interpretation, but there is no clear experimental evidence that supports it.

We agree with the reviewer that adding more readouts could provide further insights into the pattern of TORC1/PKA activity, and hopefully also give some clues on what happens in G2. We had

© 2022. Published by The Company of Biologists under the terms of the Creative Commons Attribution License (https://creativecommons.org/licenses/by/4.0/).

already considered Maf1 before, and we had carried out preliminary experiments to monitor its localization in response to rapamycin, MSX and CHX. Although Maf1 responded as expected in those experiments, we did not continue using this protein because its cytosolic localization mechanism seems to operate (at least to some degree) independently of its activity (1), which is what TORC1 and PKA mostly seem to control (2,3). This means, for example, that nuclear Maf1 can either be active or inactive, with no way of telling the activity status based on localization. Moreover, casein kinase II has also been shown to regulate Maf1 phosphorylation (4). Therefore, although we observed that Maf1 translocates as expected in response to chemical perturbations, we could not be certain if smaller moves during the cell cycle would correctly reflect changes in TORC1/PKA activity.

We additionally tested fluorescently tagged Stb3, which also responded as expected to TORC1 perturbations. However, Stb3 was almost fully cytosolic in the growth conditions that we used (Fig. S2A), which meant that we would not be able to observe bidirectional changes in TORC1 activity using this readout.

Finally, we turned to mNeonGreen-tagged Dot6, and verified that it responds correctly and strongly to rapamycin and MSX. (**Fig.S2D,E**). Note that the CHX response (**Fig.S2F)** of Dot6 appears much weaker than the Tod6 response, because the average N/C ratio of Tod6 is around 2.2-2.5, while that of Dot6 is around 1.7 (Fig. S2A). Therefore, being considerably less nuclear than Tod6, the relative drop in the Dot6 N/C ratio caused by CHX is necessarily smaller.

The cell cycle localization profile of Dot6 showed a pattern that is very good agreement with the Tod6 pattern during G1, budding and late mitosis, and anti-correlates with the Sfp1 pattern in those phases (**Fig.S5C,D**). Interestingly, Dot6 localization during much of G2 stays low, contrary to that of Tod6 which increases gradually. This behavior is in better agreement with the Sfp1 dynamics in G2, though still some divergence is still present. Overall, the Dot6 experiments further support our findings on TORC1/PKA activity outside G2. However, we did not continue with inserting Dot6 in our mutant strains, since the doubling time and volume at budding of the Dot6-mNG strain deviated too far from those of the wild-type (**Fig.S2B,C**). These observations suggested that protein tagging causes non-negligible changes in the physiology of this strain, and these changes would complicate the interpretation of comparisons between the wild type and mutant backgrounds.

3) It could be useful to distinguish between mother and daughter cells when performing the synchronization of temporal signals. Substantial differences in mother vs daughter G1 durations may blur the average dynamics of the localization of Tod6/Sfp1, and might therefore change the interpretation of the data.

We fully agree that mixing daughter and mother cell cycle patterns should be completely avoided because G1 durations vary substantially. For this reason, all synchronized signals presented in our manuscript have been derived from mother cells, i.e. cells that have budded at least once before being tracked. We tried to make this point clear in the figure captions and the main text.

4) This work provides interesting data confirming the peak of synthesis in G1 phase previously established by several groups (including the authors) and suggests that there is another peak of synthesis in G2/M. It would be interesting for the authors to discuss the relevance and significance of this second peak and why it has not been observed in their previous study (Litsios et al, 2019)

The similarity of the pTEF1-sfGFP synthesis peak in G1 (5) with the RP synthesis peak during G1 is certainly interesting. However, it should be noted that RP expression is certainly regulated, whereas TEF1 is generally considered to be constitutive (though our unpublished observations suggest that this is very likely not the case). One could speculate that the pTEF1-sfGFP and RP synthesis peaks have the same underlying cause (TORC1 dynamics), but this connection would require a separate study to establish. For these reasons, and given our current state of knowledge, we avoided connecting the two observations in our discussion.

© 2022. Published by The Company of Biologists under the terms of the Creative Commons Attribution License (https://creativecommons.org/licenses/by/4.0/).

Minor comments:

1) please show single-cell traces along with raw images corresponding to experiments in Fig 1C- J

Though we tried several options, we could not find a way to display the means together with the single-cell data without cluttering the plot. For this reason, we present the single-cell traces as heatmaps on **Fig.S3**. We hope that this type of data presentation is sufficiently clear.

2) please display consistent Y scales to appreciate differences in amplitudes on Fig 1, especially since CHX induces an effect opposite to other drugs.

Unfortunately, it is difficult to show all responses on a single scale, since they move in different directions. This opposite movement creates problems with the visualization, because upward and downward changes of the same magnitude do not look the same (for example, a twofold increase is clearer than a twofold decrease). For this reason, we had to adjust the y-axis specifically for the CHX responses, which go in the opposite direction from the rest. In any case, the main goal of Fig.1 is to show that Sfp1 and Tod6 respond in a manner that is qualitatively consistent with changes in TORC1 and PKA activity. We hope that the current plot scales do not detract from this point.

3) please indicate the strain background on each panel if different between Fig. 1C-E and 1F

We have **indicated that panels 1F and 1J correspond to the PKA-as background.**

4) Fig 3C: please show the statistical test to confirm the significance of the difference between mutants

We **included this comparison in the figure caption.**

5) On Fig 4, the Tod6-related panels are on the left column, yet they are on the right column on Fig 5. It would be more clear to have a consistent layout of the panels.

We have **modified the panel arrangement.**

6) "At the same time" on page 16: "the" is missing

It has been corrected.

7) Fig. 6: statistical tests are missing. Quantification of variability is missing (CV values, or even better, Fano factor would be necessary to compare variability in cell size since distributions have strongly different mean values)

As discussed below, we have removed the section which included Fig.6 following the comment of Reviewer 3. Some data from that figure are now presented in **Fig.S10**, along with statistical comparisons.

8) Fig. 7: Sequence of images of cells at different time points is needed to assess the fluorescence signal and the coordination with the cell cycle.

Please note that what we plotted in Fig. 7 (now Fig. 6 in the revised manuscript) is the synthesis rate of ribosomal proteins, not their concentration. It is actually very difficult to infer the synthesis rate of a stable protein from visual inspection of microscopy images, since the synthesis rate is calculated by differentiating protein abundance, which in turn depends on protein concentration (average cell fluorescence) and cell volume (mother+bud). For this reason, visually estimating the protein synthesis rate from microscopy images is extremely difficult (if not impossible).

9) Fig S2 should come before S1, because it described the methodology described in Figure S1

We have **re-ordered the supplementary figures.**

© 2022. Published by The Company of Biologists under the terms of the Creative Commons Attribution License (https://creativecommons.org/licenses/by/4.0/).

10) Fig 2A: Please provide images corresponding to fluorescently tagged Sfp1 and Tod6 on this sequence of images

Example images are provided on **Fig. S4A,B**.

11) Page 9: "all features of the wild-type Tod6 N/C ratio during G1, budding and karyokinesis had disappeared": it's a bit difficult to compare "features" by visual inspection of the data, knowing that statistical significance is not provided

We shared the concern of the reviewer, but applying classical tests such as the t-test or Mann-Whitney to the time series data of N/C ratios would not be correct, since the data at different time points come from the same cell population and are therefore correlated. To the best of our knowledge, available methods for comparing groups of time series are not appropriate in the case of our data, since these methods make very restrictive statistical assumptions about the data-generating process (e.g. stationarity, autogregressive modeling), which are violated in our case.

We therefore devised a new method for carrying out this comparison, based on modeling of our time series data using a combination of a flexible parametric model and a Gaussian process. Our method compares two sets of measured time series in order to answer the question whether they appear to come from the same underlying population. At the same time, it provides a decomposition of the total effect size over a coarse grid of time points, which allows one to visually determine the set of time points which contribute the most to the observed differences between the groups of time series. We present our method and the resulting comparisons of N/C ratios in **Supplementary Methods** and in **Figs.S12-S16**. We hope that these results will provide a more quantitative basis for comparing the N/C time series of the wild-type and the different Sfp1 and Tod6 mutants.

Reviewer 3

1. Sfp1 nuclear localization seems to be maximal around Start. The progressive increase during G1 fits with the notion proposed by Litsios et al (2019) that an autonomous metabolic cycle has an important role in execution of Start by stimulating Cln3 synthesis. In this regard, the authors state that "our results may therefore explain the cause of the observed pulses in protein synthesis and Cln3 concentration during G1". However, this statement would require a correlative analysis of Sfp1 with Whi5 localization from birth to budding. Also, the behavior of Sfp1 localization should be tested in genetically G1 arrested cells to rule out Cdk-dependent effects

Following the suggestion of the reviewer, we tracked the localization dynamics of Sfp1-GFP and Whi5-mCherry in single cells, focusing in the period just before cytokinesis and up to the next budding (i.e. the end of mitosis and subsequent G1) of mother cells. As has been observed before, Whi5 enters the nucleus a few minutes before cytokinesis, and stays there during G1, until mother cells have passed Start (6). Looking at a measure of time series similarity (cross-correlation) between the Whi5 and Sfp1 localization time series in individual cells, we saw that the Whi5 peak on average precedes the Sfp1 peak by 5-10', which means that Sfp1 peaks in the nucleus while Whi5 is exiting. We have added these observations to the main text (first section of Results) and in the Supplement (**Fig.S4**). The observation that the Whi5 peak precedes that of Sfp1 is in line with the fact that Sfp1 seems to peak around the time that Cln3 peaks in mother cells (cf. (5), Fig.6), i.e. shortly after cytokinesis, as we already pointed out in our discussion. However, we should stress that these observations are only correlative in nature, and do not provide any indications on causal connections between Whi5/Cln3 and Sfp1. Studying these connections in depth will be very interesting, in light of recent work (Albert et al., *Genes & Development*, 2019) which has shown that Sfp1 binds to many promoters of G1/S regulon genes.

2. The authors clearly show that changes in Tod6 localization during G2/M do not depend on TORC1 or PKA activity. Furthermore, Spf1 and Tod6 N/C ratios show complementary patterns in G1, but similar in G2/M, suggesting that nutrient-independent and perhaps Cdk-dependent signals operate on the mechanisms that control their localization during G2/M. This key question could be easily tested in a cdc28-4 mutant, perhaps using cdc20-arrested cells for comparison.

© 2022. Published by The Company of Biologists under the terms of the Creative Commons Attribution License (https://creativecommons.org/licenses/by/4.0/).

Given the Sfp1 and Tod6 respond very sensitively to any perturbation that affects cell growth (something we have witnessed many times in the course of our work), we were skeptical of using temperature-sensitive mutants to monitor Sfp1/Tod6 dynamics, as it would be challenging to disentangle the impact of temperature perturbations on TORC1/PKA via CDK, versus their direct impact on TORC1/PKA. To test whether CDK could be involved in the regulation of Sfp1, we therefore explored an alternative strategy, making use of a mutant with an analog-sensitive Cdc28 (Cdc28-as). Our plan was to compare the response of Sfp1 localization to the addition of 1-NM-PP1 in wild-type and in Cdc28-as cells. If Cdc28 is involved in the regulation of Sfp1 localization, one should see a stronger Sfp1 response in Cdc28-as cells soon after the addition of 1-NM-PP1.

Unfortunately, the experiments did not work out as we hoped. First, Cdc28-as cells were more elongated than wild-type and divided more slowly in the absence of 1-NM-PP1, which indicated that Cdc28 activity was already compromised to some extent, and therefore pre-treatment conditions were not the same in wild-type and Cdc28-as cells. Second, the high concentration of 1-NM-PP1 (5 μM) needed to inhibit Cdc28 activity had quite strong off-target effects in wild-type cells. Consequently, the short-term responses of Sfp1 in the Cdc28-as background and the wild-type were indistinguishable from each other, as shown in the figure below. Note that due to the lack of a nuclear marker in those cells, the standard deviation of the GFP signal over the cell area was used as a proxy of nuclear localization. As we verified in (7), the standard deviation correlates well with the N/C ratio, and can capture large changes in protein localization, such as those induced by chemical inhibitors.

NOTE: *We have removed unpublished data that had been provided for the referees in confidence.*

On longer time scales, it is interesting to observe that the nuclear localization of Sfp1 recovers in Cdc28-as cells, whereas it does not do so in the wild-type. Furthermore, Cdc28-as cells arrest upon treatment with 1-NM-PP1, whereas the wild-type does not, as we verified by examining the microscopy movies. Therefore, the recovery of Sfp1 nuclear localization in 1-NM-PP1-treated Cdc28-as cells is an indication that Cdc28 activity is not required to maintain Sfp1 in the nucleus. However, the coordination between TORC1 activity and the cell cycle is lost in those cells, and it is also possible that TORC1 activity somehow increases under those conditions. Overall, our tests with Cdc28-as did not allow us to draw concrete conclusions about the direct effects of Cdc28 on Sfp1.

3. Bcy1 mutant cells are much larger, which likely explains the observed differences in volume growth rate when compared to wt. The same reasoning in the opposite sense applies to pib2 cells. Thus, the conclusion that "alterations in the TORC1 and PKA cell cycle activity pattern perturb the coupling between cell cycle and growth" is not supported by these data, which should be removed or complemented by independent approaches.

We understand that the terminology used in the section describing the growth of mutants was a bit too vague, and that there are several ways of looking at the volume dynamics. For example, one could plot the specific growth rate (i.e. volume increase rate divided by volume), in which case the differences between wild-type and mutants are minimized. Which growth metric is more relevant in this particular case, is not clear to us. On the other hand, it remains true that the mutants bud and divide at different sizes, which indicates that the coupling between the cell cycle and cell size has been perturbed in these cells (our use of “growth” in place of “size” in the original statement was incorrect), and that their target size has shifted. In any case, given the difficulties in interpreting the growth data, we decided to follow a more conservative (and, hopefully, less confusing for the readers) approach, by completely removing the section on growth dynamics and incorporating some of the volume observations in the preceding section, where the mutants are first described.

4. RP synthesis patterns are interesting and coherent with the observed changes in Spf1 and Tod6 localization. Rate values are plotted per volume unit, which is correct but does not reflect the rate change in volume growth. I would suggest the authors add a plot with the overall rate data.

© 2022. Published by The Company of Biologists under the terms of the Creative Commons Attribution License (https://creativecommons.org/licenses/by/4.0/).

We have added the corresponding plots to **Fig. S11**.

References

1. Wei Y, Tsang CK, Zheng XFS. Mechanisms of regulation of RNA polymerase III-dependent transcription by TORC1. EMBO J. 2009;28(15):2220–30.
2. Boguta M. Maf1, a general negative regulator of RNA polymerase III in yeast. Biochim Biophys Acta - Gene Regul Mech. 2013;1829(3–4):376–84.
3. Wei Y, Zheng XFS. Maf1 regulation. Nucleus. 2010;1(2):162–5.
4. Graczyk D, Dębski J, Muszyńska G, Bretner M, Lefebvre O, Boguta M. Casein kinase II-mediated phosphorylation of general repressor Maf1 triggers RNA polymerase III activation. Proc Natl Acad Sci U S A. 2011;108(12).
5. Litsios A, Huberts DHEW, Terpstra HM, Guerra P, Schmidt A, Buczak K, et al. Differential scaling between G1 protein production and cell size dynamics promotes commitment to the cell division cycle in budding yeast. Nat Cell Biol. 2019 Mar 8;21(11):1382–92.
6. Costanzo M, Nishikawa JL, Tang X, Millman JS, Schub O, Breitkreuz K, et al. CDK activity antagonizes Whi5, an inhibitor of G1/S transcription in yeast. Cell. 2004;117(7):899–913.
7. Litsios A, Huberts DHEW, Terpstra HM, Guerra P, Schmidt A, Buczak K, et al. Differential scaling between G1 protein production and cell size dynamics promotes commitment to the cell division cycle in budding yeast. Nat Cell Biol. 2019;21(11):1382–92.

Original submission

First decision letter

MS ID#: JOCES/2022/260378

MS TITLE: TORC1 and PKA activity towards ribosome biogenesis oscillates in synchrony with the budding yeast cell cycle

AUTHORS: Paolo Guerra, Luc-Alban Vuillemenot, Yulan B van Oppen, Marije Been, and Andreas Milias-Argeitis
ARTICLE TYPE: Research Article

Thank you for sending your manuscript to Journal of Cell Science through Review Commons.

I am happy to tell you that your manuscript has been accepted for publication in Journal of Cell Science, pending standard ethics checks.

© 2022. Published by The Company of Biologists under the terms of the Creative Commons Attribution License (https://creativecommons.org/licenses/by/4.0/).
